# Subsurface Oxygen Vacancy Mediated Surface Reconstruction and Depolarization of Ferroelectric BaTiO_3_ (001) Surface

**DOI:** 10.1002/advs.202412781

**Published:** 2025-02-13

**Authors:** Jeehun Jeong, Jaejin Hwang, Yaolong Xing, Zhipeng Wang, Jaekwang Lee, Sang Ho Oh

**Affiliations:** ^1^ Department of Energy Engineering KENTECH Institute for Energy Materials and Devices Korea Institute of Energy Technology (KENTECH) Naju 58330 Republic of Korea; ^2^ Department of Physics Pusan National University Busan 46241 Republic of Korea; ^3^ Center for Shared Research Facilities Korea Institute of Energy Technology (KENTECH) Naju 58330 Republic of Korea

**Keywords:** depolarization, ferroelectrics, in‐situ high‐resolution transmission electron microscopy, perovskite oxides, surface reconstructions

## Abstract

The interplay between surface reconstruction and depolarization of ferroelectric oxide surfaces is strongly influenced by oxygen vacancies (V_O_). Using in‐situ atomic‐resolution electron microscopy imaging and spectroscopy techniques, it is directly observed that a clean BaTiO_3_ (001) surface stabilizes into (2 × 1) BaO‐terminated reconstruction during vacuum annealing. This surface reconstruction is achieved with accommodating BaO deficiency and incorporates TiO_x_ adunits. The cooperative atomic rumpling in both the surface and subsurface layers, arranged in a tail‐to‐tail configuration, is stabilized by planar accumulation of V_O_ in the subsurface TiO_2_ layer. This reduces the net polarization of surface unit cells, contributing to overall depolarization. Under this atomic rumpling, the polarization‐down (P↓) state is energetically favored over the polarization‐up (P↑) state, as the P↓ state requires less atomic relaxation in the bulk layers to achieve dipole inversion at the subsurface. The energetic preference for V_O_ in the subsurface TiO_2_ layer of the P↓ state is confirmed through calculations of V_O_ formation energy and the energy barrier for surface‐to‐subsurface migration. These findings reveal that the presence of V_O_ in the subsurface layer lifts the degeneracy in the double‐well potential between the P↓ and P↑ states in BaTiO_3_ (001).

## Graphical abstract

In‐situ atomic‐scale electron microscopy reveals that vacuum‐annealed ferroelectric BaTiO_3_ (001) surfaces exhibit BaO termination with periodic Ba deficiency and TiO_x_ adunits. Planar oxygen vacancy accumulation in the subsurface TiO_2_ layer mitigates the depolarization field, facilitating cooperative rumpling of surface and subsurface layers in a tail‐to‐tail dipole configuration, where downward polarization is relatively more stable than upward polarization.

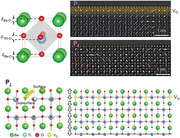

## 1 Introduction

Ferroelectrics, known for their switchable spontaneous polarization below the Curie temperature (*T_C_*), play a crucial role in a wide range of technological applications. For example, switchable polarization is essential for devices like nonvolatile memory storage, capacitors, and piezoelectric sensors.^[^ ^1^, ^2^ ^]^ As devices continues to shrink, the precise control of the polarization, especially at the nanoscale, becomes increasingly vital.^[^ ^3^, ^4^, ^5^, ^6^ ^]^ A key challenge is ensuring that polarization remains stable up to the boundaries of a system, such as free surface or interfaces with other materials.^[^ ^7^, ^8^, ^9^, ^10^ ^]^ However, maintaining and controlling ferroelectricity at reduced dimensions faces significant challenges due to unavoidable depolarization effects.^[^ ^11^, ^12^ ^]^

Polarization in ferroelectrics can remain stable up to the surface without depolarization when external charges, such as free electrons from adjacent metal electrodes or polar adsorbates from the atmosphere, are present.^[^ ^13^, ^14^ ^]^ In the absence of external charges, polarization gradually diminishes toward the surface or rotates into a closed vortex to minimize charges from polarization gradients.^[^ ^7^, ^15^, ^16^ ^]^ These depolarization effects render ferroelectric surfaces inactive and can dominate the net polarization of the entire system as its size approaches the nanometer scale.^[^ ^11^, ^17^ ^]^

Depolarization of ferroelectric surfaces often involves the displacement of cations and anions, leading to the rumpling of surface atomic layers^[^ ^18^, ^19^ ^]^ and an ionic polarization that counteracts the intrinsic polarization. Therefore, in clean, single‐crystalline ferroelectric surfaces, depolarization is closely tied to surface relaxation or reconstruction, where an ionic polarization evolves within the framework of surface reconstruction that is stabilized under a specific environmental condition.^[^ ^20^, ^21^, ^22^ ^]^ It is important to note that clean and atomically smooth surfaces, essential for surface science or thin film growth, are typically prepared through high‐temperature annealing in a vacuum.^[^ ^18^, ^23^, ^24^, ^25^, ^26^, ^27^, ^28^, ^29^ ^]^ Under such annealing conditions, ferroelectric oxide surfaces tend to become oxygen‐deficient due to the spontaneous formation of oxygen vacancies (V_O_), which actively participate in both surface depolarization and reconstruction processes.^[^ ^13^, ^18^, ^30^ ^]^ For instance, atomistic calculations predict that the lattice distortion around V_O_, coupled with its electrostatic interactions with neighboring atoms, favors specific polarization orientations.^[^ ^31^, ^32^, ^33^, ^34^, ^35^, ^36^ ^]^ Additionally, electron doping via V_O_ can induce 2D electrical conductivity,^[^ ^37^, ^38^, ^39^ ^]^ which in turn affects polarization stability at the surface.

Barium titanate (BaTiO_3_), a prototypical lead‐free ferroelectric oxide, is widely used for electronic devices such as multilayer ceramic capacitors and ferroelectric tunnel junctions.^[^ ^40^, ^41^, ^42^, ^43^, ^44^, ^45^ ^]^ At high temperatures, BaTiO_3_ adopts a cubic (*Pm* $\bar{3}$ *m*) phase, and upon cooling below the *T_C_* (≈125 °C), it undergoes the phase transition to a tetragonal phase (*P4* *mm*) with its polar axis aligned along the <001> direction.^[^ ^46^ ^]^ Theoretical phase diagrams calculated for tetragonal BaTiO_3_ (001) surfaces predict various surface reconstructions as a function of the chemical potential.^[^ ^21^, ^24^, ^47^, ^48^, ^49^ ^]^ When polarization is included for the surface energy calculation, the (2 × 1) reconstruction becomes more stable in the polarization‐down (P↓) configuration across a broad range of chemical potentials, while the polarization‐up (P↑) configuration becomes unstable unless the oxygen chemical potential is very low.^[^ ^49^, ^50^ ^]^ Experiments have shown that BaTiO_3_ (001) surface annealed in oxygen‐deficient conditions stabilizes into either a (1 × 1) BaO reconstruction^[^ ^18^, ^51^ ^]^ or a (2 × 1) double TiO_2_ reconstruction,^[^ ^24^, ^48^, ^49^, ^50^ ^]^ depending on annealing conditions. Although the surface chemistry of vacuum‐annealed BaTiO_3_ (001) has been characterized in most experimental studies, its accuracy is still debated due to inherent limitations in model‐based interpretation of experimental data. Additionally, the distribution of V_O_ and its effects on surface reconstruction and depolarization remain underexplored.

In this study, in‐situ, atomic‐scale electron microscopy imaging and spectroscopy analysis reveal that the BaTiO_3_ (001) surface stabilizes into a (2 × 1) reconstruction under vacuum annealing conditions. This surface reconstruction is achieved on the BaO termination layer with involving periodic Ba deficiency and TiO_x_ adunits. Importantly, the subsurface TiO_2_ layer beneath this reconstructed surface accommodates V_O_ that play a pivotal role in compensating the depolarization field and stabilizing polarization. The cooperative rumpling of the surface and subsurface layers, coupled with the planar accumulation of V_O_ in the subsurface, forms a tail‐to‐tail dipole configuration that reduces net polarization. This mechanism energetically favors the polarization‐down (P↓) state and mitigates depolarization effects at the nanoscale.

## 2 Results and Discussion

### 2.1 Surface Termination and Reconstruction

**Figure 1:**
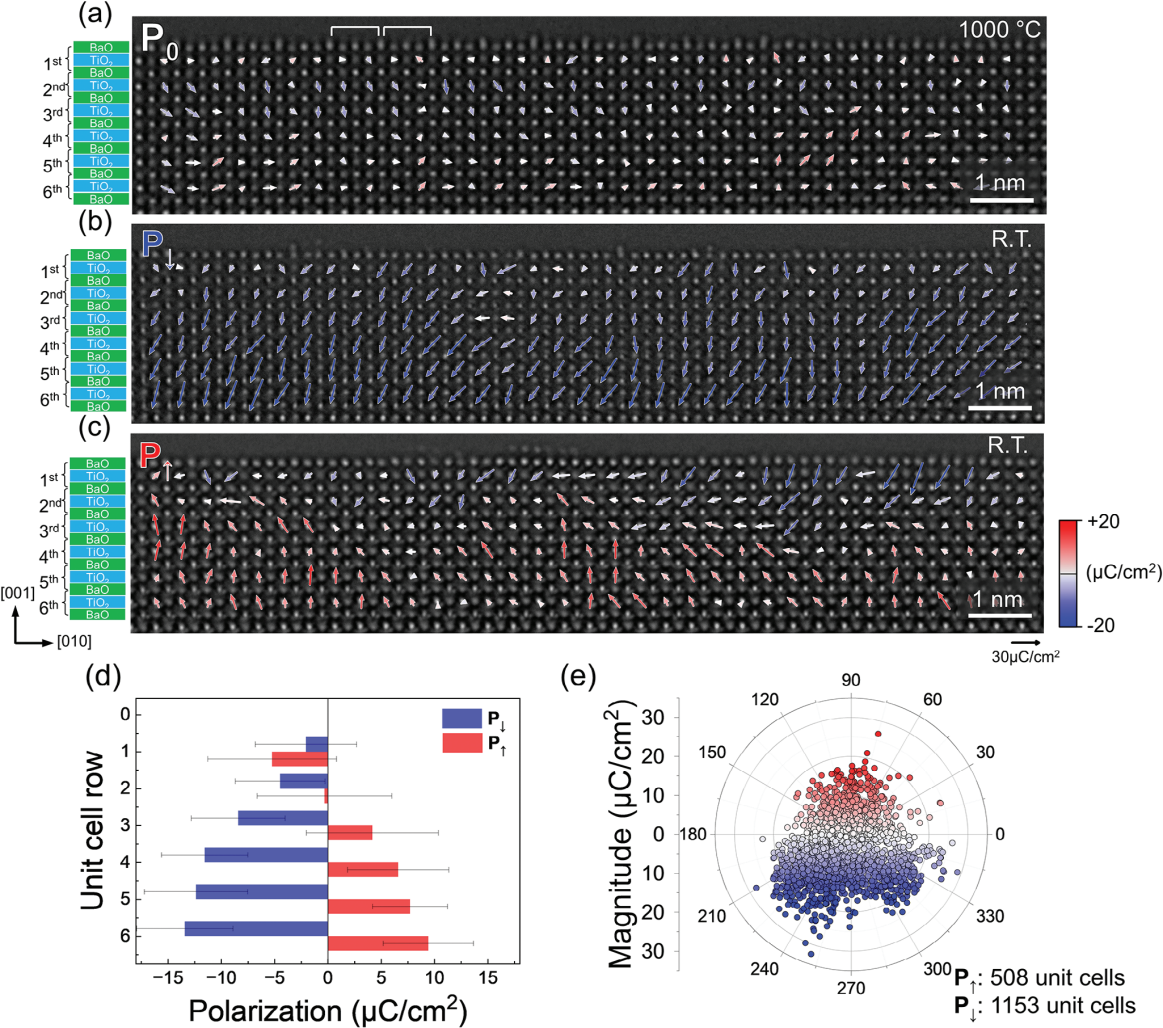
Polarization vector maps of clean vacuum‐annealed BaTiO_3_ (001) surfaces. The polarization vector of each unit cell was determined by measuring the atomic displacements directly on high‐resolution TEM (HRTEM) images recorded at a negative Cs imaging condition (NCSI). Details for the measurement are given in the Methods. a) Polarization map (P_0_) of the cubic phase at 1000 °C. The results are used to set the lower bound of measurable polarization by the current method, which is ±4 µC cm^−2^ (Figure S5, Supporting Information). The surface is terminated by the BaO layer as determined by STEM imaging and EELS elemental mapping (Figures S2,S3, Supporting Information). The two‐unit cell periodicity corresponding to the (2 × 1) surface reconstruction is indicated by white lines. The (2 × 1) reconstruction was confirmed by plan‐view electron diffraction pattern (Figure S1, Supporting Information). Polarization maps of the tetragonal phase in polarization‐down P↓ (b) and polarization‐up P↑ (c) state at room temperature (R.T.). In both P↓ and P↑ states the polarization decreases toward the (001) surface. In the P↑ state the polarization vectors in‐between the (upward) bulk to the (downward) surface polarization tend to rotate toward the in‐plane directions. d) Averaged polarization of each unit cell row from the bulk to the surface in P↓ (blue) and P↑ (red) states. The error bars indicate the standard deviation. The inversion of the polarization vector from up to down is observed near the surface of the P↑ state. e) Polar plot of the measured polarization vectors. The population of P↓ domains is almost twice that of P↓ domains. Some of the polarization vector maps used for this plot are provided in Figure S6 (Supporting Information).

A clean, atomically smooth BaTiO_3_ (001) surface in an edge‐on orientation was obtained by annealing a transmission electron microscopy (TEM) sample at 1000 °C for one hour in the vacuum environment (≈2 × 10⁻⁵ Pa) of a TEM column. This vacuum annealing effectively removed surface contaminants and damages induced by focused ion beam (FIB) milling.^[^ ^26^ ^]^ Under these annealing conditions, the cleaned BaTiO_3_ (001) surface underwent layer‐by‐layer evaporation with advancing atomic steps, exposing a freshly terminated surface in a step‐terrace structure (Movie S1, Supporting Information). The atomically smooth terraces, separated by atomic steps with one‐unit cell (u.c.) height, were consistently terminated by a BaO layer that stabilized into a (2 × 1) reconstruction (**Figure** **1**a; Figure S1a,b, Supporting Information). Among the various surface reconstructions predicted in the phase diagrams,^[^ ^24^, ^49^ ^]^ this type of (2 × 1) reconstruction has been observed most frequently for the BaTiO_3_ (001) surface annealed at temperatures above 700 °C in vacuum.^[^ ^50^ ^]^

While the (2 × 1) reconstruction forms on an apparent BaO termination, its surface chemistry deviates from the exact stoichiometry of BaO (Figure S2, Supporting Information). In‐situ atomic‐resolution electron energy loss spectroscopy (EELS) elemental mapping revealed consistently low Ba occupancy in the surface BaO layer every two u.c., accompanied by additional Ti atoms occupying nearby octahedral (O_h_) sites where Ba atoms were missing (Figure S3, Supporting Information). The outermost Ti atoms, repeating every two u.c., produced a characteristic rumpling pattern of the surface layer. The coexistence of Ba and Ti in the surface layer, alongside their unique (2 × 1) reconstruction, can be attributed to the different thermal behaviors of the two binary oxides, BaO and TiO_2_, during the evaporation of BaTiO_3_ (001) surface via step migration. Due to the preferential evaporation of BaO,^[^ ^24^ ^]^ the freshly terminated BaO surface becomes a Ba‐deficient state as some portion of Ba is removed along with the advancing atomic step. This Ba‐deficient surface is unstable, prompting the diffusion of TiO_2_ from the moving step edge toward the surface, where it arranges into stable (2 × 1) reconstruction (Movies S2,S3, Supporting Information).

The deficiency of Ba in the BaO termination layer is consistent with previous X‐ray photoelectron spectroscopy studies,^[^ ^18^, ^52^, ^53^, ^54^, ^55^ ^]^ which observed the presence of Ba vacancies with lowered oxygen coordination on vacuum‐annealed BaTiO_3_ (001) surfaces. However, the presence of TiO_x_ on Ba‐deficient BaO surfaces and its role in the (2 × 1) reconstruction has not been observed experimentally nor considered theoretically until now. Notably, the previously reported (2 × 1) reconstruction, claimed to be thermodynamically stable, was based on a fully occupied TiO_2_ surface layer, where additional TiO_x_ adunits occupied hollow sites on the TiO_2_ layer.^[^ ^49^, ^50^ ^]^ The reason why the previous experimental studies failed identifying the surface chemistry correctly is likely due to the limitations of model‐based interpretations of X‐ray surface diffraction or spectroscopy data, which typically explore a narrow parameter space. A similar non‐stoichiometric (2 × 1) reconstruction has also been observed for vacuum‐annealed SrTiO_3_ (001) surfaces, suggesting that this type of surface reconstruction may be a common feature among other perovskite oxides.^[^ ^23^ ^]^ This discovery underscores the need for further in‐depth theoretical studies to investigate the mechanism behind non‐stoichiometric (2 × 1) reconstructions.

As the temperature decreased from the annealing condition, the characteristic rumpling pattern of the (2 × 1) reconstruction became less pronounced (Movie S4, Figure S4, Supporting Information). At room temperature, although Ti atoms remained at the octahedral sites in the surface layer, the (2 × 1) reconstruction was no longer maintained over long ranges, as indicated by the extinction of super‐reflections in the electron diffraction pattern (Figures S1c,S4, Supporting Information). Additionally, a few isolated TiO_x_ adunits, frozen from surface diffusion during cooling, were observed decorating the surface.

### 2.2 Surface Polarization

Below the *T_C_*, BaTiO_3_ transforms to the tetragonal phase which possesses spontaneous polarization (P) along its <001> polar axis.^[^ ^46^ ^]^ To investigate how the polarization is modified near the (001) surface to compensate the depolarization field, polarization was measured unit cell‐by‐unit cell from the bulk to the surface using high‐resolution TEM (HRTEM) images. The polarization was determined by measuring the displacement of Ti and O atoms from the centrosymmetric position of the Ba sublattice (Figure S5, Supporting Information). For simplicity, the formal ionic charges were used in the calculations. The precision of the measurements was tested on the cubic BaTiO_3_ phase under the annealing conditions (Figure 1a; Figure S5, Supporting Information). The measured displacements were within ±4 pm, corresponding to a polarization of ≈±4 µC cm^−2^, setting the lower bound for measurable polarization with this method. The polarization measured for surface unit cells, including the (2 × 1) reconstructed surface layer, remained similar to that of the bulk BaTiO_3_. This is because the large positive rumpling of the reconstructed surface layer is counterbalanced by the negative rumpling of the subsurface TiO_2_ layer. This cooperative relaxation underscores the crucial role of subsurface relaxation in compensating any dipole moments arising from surface reconstruction.

In the tetragonal BaTiO_3_ phase stabilized at room temperature, ferroelectric domains with polarization aligned along the [001] and [00$\bar{1}$] directions, referred to as polarization‐up (P↑) and polarization‐down (P↓), were clearly resolved (Figure 1b,c). As it moves closer to the (001) surface, polarization gradually decreases in both P↑ and P↓ domains, effectively reducing the depolarization field (Figure 1d). The persistence of a small but finite polarization at the surface suggests partial compensation of the depolarization field through other mechanisms, such as domain ordering or electronic charge compensation.^[^ ^49^ ^]^ Statistical analysis of multiple polarization maps revealed that P↓ domains were almost twice as prevalent as P↑ domains (Figure 1e; Figure S6, Supporting Information). This observation aligns with previous studies reporting the dominance of P↓ domains on BaTiO_3_ surfaces annealed under reducing conditions.^[^ ^20^, ^21^ ^]^ Moreover, the mean polarization value (Figure 1e), averaged over the four unit‐cells below the surface, was greater for P↓ (≈18 µC cm^−2^) than for the P↑ (≈12 µC cm^−2^). These results suggest that high‐temperature vacuum‐annealing likely introduces an asymmetry in the double‐well potential, lifting the degeneracy of P↑ and P↓ states near the surface.

P↑ and P↓ domains exhibit distinctly different polarization behaviors near the (001) surface (Figure 1d). In P↑ domains, the surface polarization reverses from the bulk polarization, pointing downward. Another noticeable feature in P↑ domains is the rotation of polarization toward the in‐plane directions in the transition zone between bulk and surface polarization. In contrast, bulk polarization in P↓ domains persists to the surface without inversion, with only the magnitude gradually decreasing. As a result, the surface unit cells in both P↑ and P↓ domains exhibit reduced but finite downward polarization, regardless of their bulk polarization. Given that no significant differences in surface reconstruction are noticed between the P↑ and P↓ states, a clue to this unique surface depolarization behavior likely lies beneath the surface, specifically in the subsurface TiO_2_ layer. Our analysis shows that V_O_ accumulated in the subsurface TiO_2_ layer plays a pivotal role in stabilizing the reduced downward surface polarization by inducing specific atomic rumpling patterns in both the surface and subsurface layers. Direct evidence for V_O_ accumulation beneath the surface is provided through quantitative analysis of HRTEM images and EELS, as discussed in detail below.

### 2.3 Oxygen Vacancies at Subsurface TiO_2_

**Figure 2:**
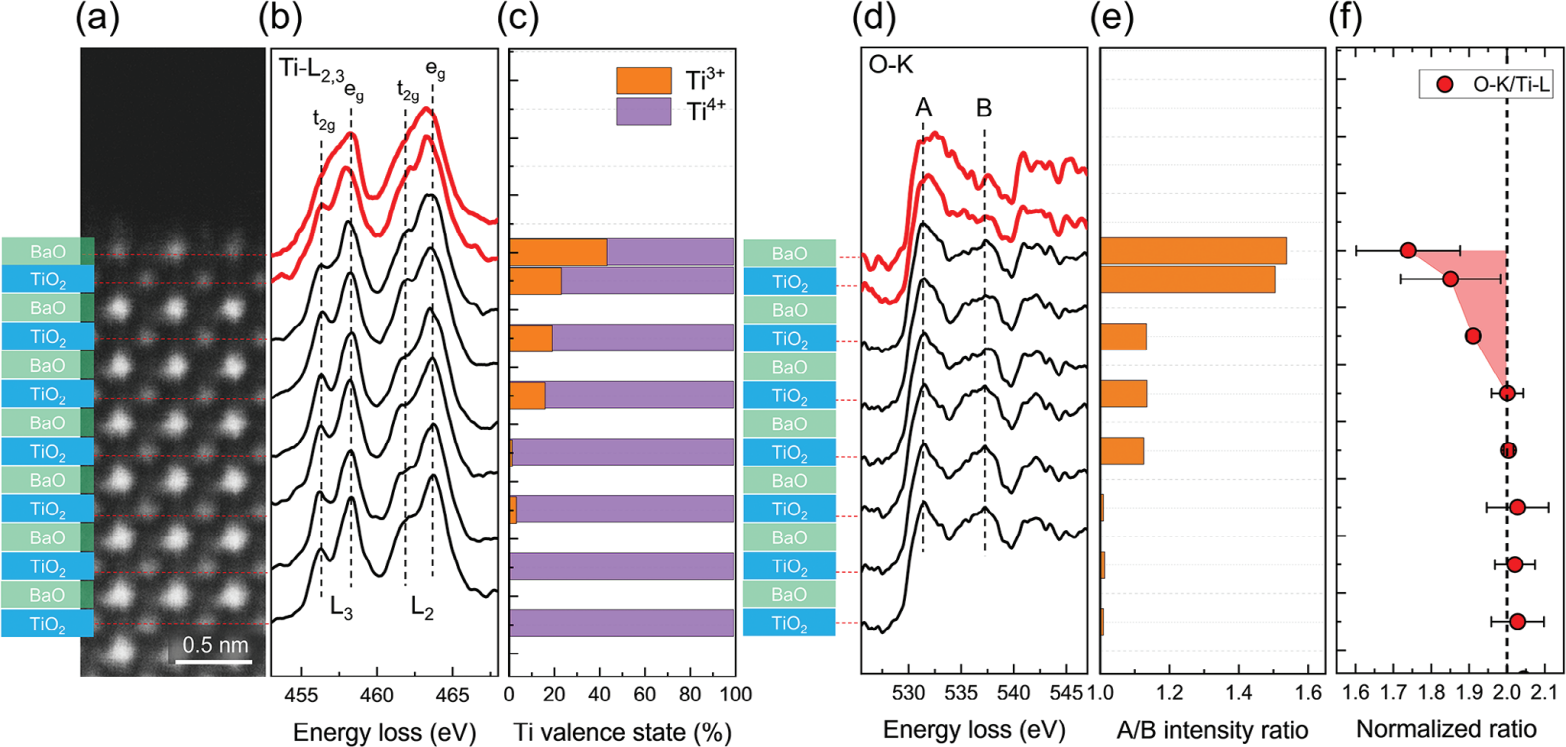
Oxygen vacancies (V_O_) formed at the surface and subsurface layers of vacuum‐annealed BaTiO_3_ (001) detected by STEM EELS. a) ADF STEM survey image acquired for EELS measurement at 700 °C. The BaO and TiO_2_ layers are labeled from the bulk to the surface. Ti‐L_2,3_ and O‐K edge EEL spectra acquired from each TiO_2_ layer from the bulk to the surface (including the surface layer) are displayed. b) Ti‐L_2,3_ edge EEL spectra. The fine structure of the surface and subsurface EEL spectra (highlighted in red) is modified by the presence of the Ti^3+^ state. c) Ti^3+^/Ti^4+^ ratio determined by multiple linear least square fitting (MLLS) of the Ti‐L_2,3_ edge EEL spectra. The reference spectra for Ti^4+^ and Ti^3+^ were obtained from bulk BaTiO_3_ from LaTiO_3_, respectively.^[^ ^59^ ^]^ d) O‐K edge EEL spectra. The first peak (labeled A) in the fine structure originates from the transition from O 1s to O 2*p*‐Ti 3*d* hybridized orbital. The second peak (labeled B) originates from the transition from O 1*s* to the O 2*p*‐Ba 5*d* hybridized orbital. The intensity of peak B is reduced in the surface and subsurface EEL spectra (highlighted in red) by the presence of V_O_. e) Intensity ratio of peak A to B. The high A/B peak intensity ratios of the surface layer and subsurface TiO_2_ layer indicate the presence of V_O_. f) O/Ti ratio determined by using the integrated intensity of O‐K and Ti‐L_2,3_ edges. The O/Ti ratio smaller than the stoichiometry of 2.0, that is, oxygen deficiency, is highlighted in red.

It is well established that the BaTiO_3_ (001) surface becomes electrically conductive when annealed in a vacuum due to the formation of V_O_, which acts as electron donors.^[^ ^36^, ^56^ ^]^ To elucidate the spatial distribution of V_O_, we analyzed the EELS Ti‐L_2,3_ and O‐K edges acquired layer‐by‐layer from the bulk to the surface (**Figure** **2**). The Ti‐L_2,3_ edges of the surface and subsurface TiO_2_ layers exhibit notable changes, indicative of the presence of Ti^3+^ state in addition to Ti^4+^ (Figure 2b). The Ti^3+^/Ti^4+^ ratio, determined through multiple linear least squares (MLLS) fitting, shows an increasing trend toward the surface, suggesting that the extra electrons donated by V_O_ occupy the Ti‐3*d* orbitals (Figure 2c).^[^ ^57^ ^]^ The O‐K edges of the corresponding layers also reveal distinct fine structures compared to the bulk reference. Most notably, there is a reduction in the intensity of the second peak (labeled B) relative to the first peak (labeled A) (Figure 2d,e). The second peak of the O‐K edge, originating from the transition from O 1*s* to the O 2*p*‐Ba 5*d* hybridized orbitals, is known to sensitive to V_O_.^[^ ^57^, ^58^ ^]^ The reduced intensity of this peak indicates a deficiency of Ba and oxygen. Additionally, the quantification of the O‐K and Ti‐L_2,3_ edges shows a decreasing O/Ti ratio from the stoichiometric value of 2.0 toward the surface (Figure 2f). Collectively, all EELS data consistently demonstrate that V_O_ is predominantly located at the BaTiO_3_ (001) surface, followed by the subsurface TiO_2_ layer, with a gradual decrease in V_O_ concentration toward the bulk.

**Figure 3:**
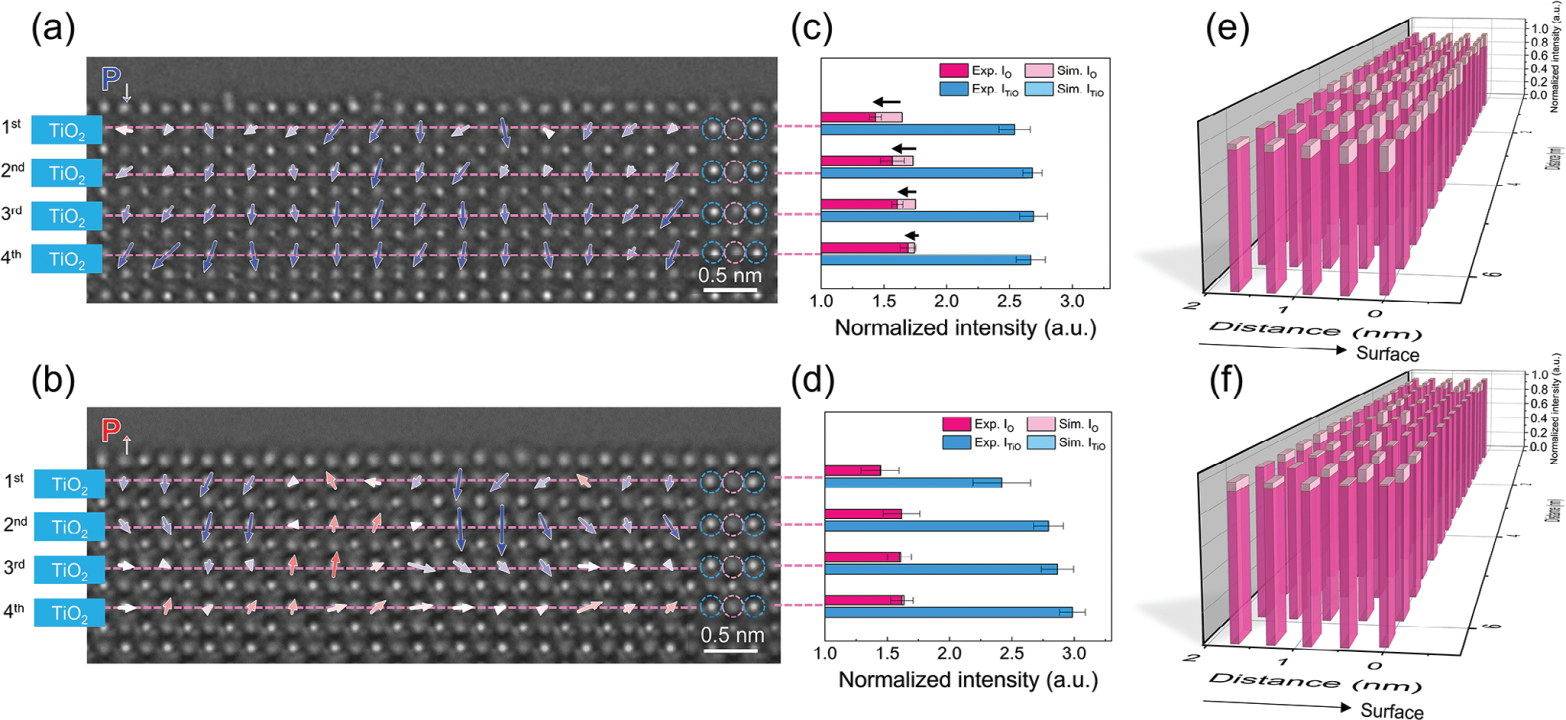
Polarization‐dependent near‐surface oxygen vacancy distribution determined by HRTEM image analysis. NCSI HRTEM image of P↓ (a) and P↑ (b) state with polarization vectors being overlaid. Dashed circles indicate the TiO (blue) and O (pink) columns in the TiO_2_ layer. The averaged intensity of TiO (blue) and O (pink) columns in each TiO_2_ layer from the bulk to the surface for P↓ (c) and P↑ (d) state. The averaged intensity TiO columns is used to determine the thickness of the TEM specimen (refer to Figures S7,S8, Supporting Information). For the given thickness of the TEM specimen, the expected intensity of the O column from the simulation is indicated by light pink. The measured intensity of the O column is overlaid on the expected one by using dark pink. Uncovered light pink by dark one indicates the deficiency of oxygen in the O column, as marked by an arrow. Normalized intensity plot of the O columns in the TiO_2_ layers of P↓ (e) and P↑ (f) state. The HRTEM images shown in (a) and (b) were used for the intensity analysis. The color coding is the same as in (c) and (d); Uncovered light pick by dark one indicates the deficiency of oxygen in the O column. The accumulation of V_O_ near the subsurface TiO_2_ layers is pronounced more in the P↓ than P↑ state.

To investigate the differences in V_O_ distribution between the P↑ and P↓ polarization states of the BaTiO_3_ (001) surface, we quantitatively analyzed the atomic column intensities of HRTEM images (**Figure** **3**). HRTEM image simulations demonstrate that the intensity of atomic columns increases linearly with the number of atoms, provided the TEM specimen thickness is less than 6 u.c. (Figures S7,S8, Supporting Information). The local thickness of the near‐surface region was determined by cross‐correlating experimental HRTEM images with simulated ones at various thicknesses, revealing the thickness ranging from 4 to 8 u.c. in most images. In the P↑ state, the intensities of all TiO and O atomic columns in the TiO_2_ layers closely match the simulated values, following the thickness variation (Figure 3b,d; Figure S7, Supporting Information). In contrast, for the P↓ state, while the TiO column intensities align well with the simulations and are used to determine the sample thickness, the O column intensities are significantly lower than the expected ones, indicating the presence of V_O_ in these columns (Figure 2a,c; Figure S8, Supporting Information).^[^ ^60^, ^61^ ^]^ The disparity between the experimental and simulated intensities of the O columns increases toward the surface. The intensity measurements across multiple unit cells reveal that, unlike the P_↑_ state (Figure 3f), the P↓ state exhibits a denser and more widespread distribution of V_O_ (Figure 3e). A pronounced deficiency in the O column occupancy extends from the subsurface TiO_2_ layer to the fourth layer, consistently across all 16‐unit cells along the in‐plane direction. This finding, which demonstrates more V_O_ in the P↓ state, combined with the fact that P↓ is observed more frequently than P↑, suggests that the P↓ state is energetically favored in the presence of V_O_ near the surface. Notably, a substantial amount of V_O_ is present not only in the surface layer but also in the subsurface TiO_2_ layer, highlighting the crucial role of the subsurface in compensating the depolarization field and accommodating the conductive electrons generated by V_O_. This effect appears to result from the collective influence of multiple V_O_ in the subsurface layer rather than individual vacancies. In fact, planar accumulation of V_O_ in the subsurface TiO_2_ layer also leads to the expansion of interlayer spacing (*d*_i,j_), not only between the surface and subsurface layers (*d*_1,2_) but also between the subsurface and the third layer (*d*_2,3_) (Figure S9, Supporting Information).

### 2.4 Tail‐to‐Tail Dipole Inversion at Subsurface

**Figure 4:**
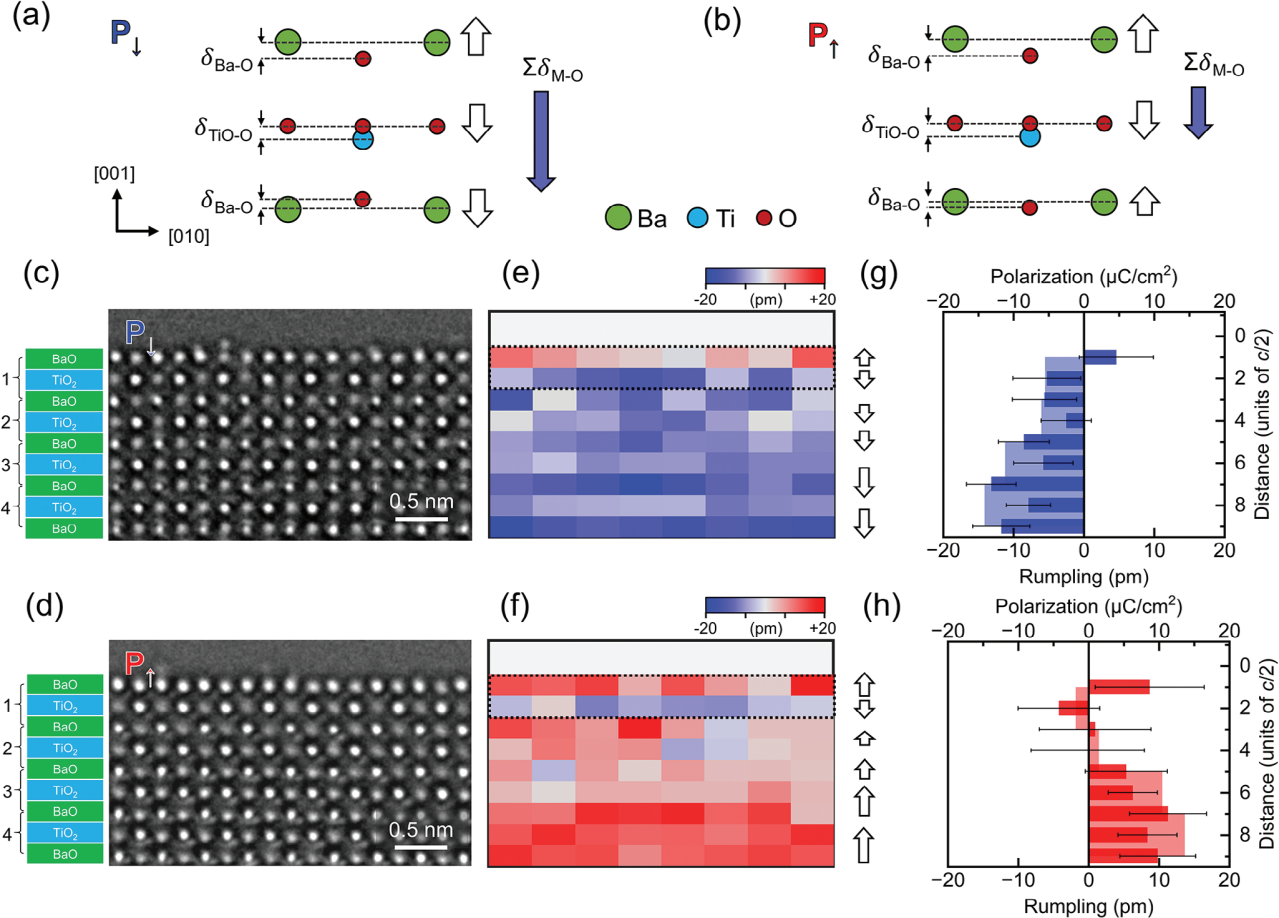
Polarization‐dependent near‐surface atomic rumpling patterns determined by HRTEM image analysis. Schematic illustration of the atomic rumpling of the surface unit cells in P↓ (a) and P↑ (b) state and their relationship with polarization. The out‐of‐plane displacement of cation from oxygen is defined as the rumpling amplitude, *d*_Ba‐O_ for BaO, *d*_Ti‐O_ for the TiO_2_ layer. The upward displacement of the cation is assigned to a positive and the downward to a negative sign. The summation of *δ*_Ti‐O_ and partitioned *δ*_Ba‐O_ (half from the upper and the other half from the lower BaO layers) is equivalent to the out‐of‐plane component of polarization. The rumpling and polarization measured by HRTEM image analysis are schematically represented by open and solid (blue) arrows, respectively. HRTEM images of P↓ (c) and P↑ (d) state used for layer‐by‐layer measurement of atomic rumpling. Atomic rumpling *d*_Ba‐O_ and *d*_Ti‐O_ maps of P↓ (e) and P↑ (f) state. The plot of rumpling amplitude *d*_Ba‐O_ and *d*_Ti‐O_ averaged along the in‐plane direction for P↓ (g) and P↑ (h) state. The error bars indicate the standard deviation. The overlaid shade color indicates the polarization of the unit cell. The tail‐to‐tail dipole inversion in the surface and subsurface layers is commonly observed in both P↓ and P↑ (refer to the arrows next to the plots), which decreases the polarization of the surface unit cell. While the surface polarization remains downward in the P↓ state, it shows inversion in the P↑ state.

The rumpling amplitude of each atomic layer, measured as the out‐of‐plane displacement of the cation relative to the oxygen (*δ*_M‐O_), represents the dipole moment induced along the polar axis (**Figure** **4**a,b).^[^ ^19^, ^36^ ^]^ Tracing the rumpling amplitude from the bulk to the surface (Figure 4c–h) reveals the detailed evolution of the dipole moment in each layer as it compensates for the depolarization field. In both P↑ and P↓ states, the rumpling of the surface layer, where the cations are displaced all above the oxygen atoms, contributes to an upward surface dipole. Beneath this surface layer, the subsurface TiO_2_ layer exhibits a downward dipole, which is similarly present in both polarization states. This configuration of antiparallel dipoles–upward in the surface layer and downward in the subsurface TiO_2_ layer–remains consistent regardless of the bulk polarization direction (P↑ or P↓). These antiphase dipoles, arranged in a tail‐to‐tail configuration, serve to reduce the net surface polarization while stabilizing the bulk polarization. Notably, in both P↑ and P↓ domains, the out‐of‐plane component of polarization in the surface unit cells points downward, as the larger dipole moment contribution from the subsurface TiO_2_ layer outweighs those from the surface and third layers.

While the upward surface dipole faces free space (vacuum), the downward subsurface dipole faces those of rigid bulk. Consequently, the surface dipole evolves in close correlation with surface reconstruction, while the subsurface dipole interacts with those of subsequent bulk layers to minimize the polarization gradient. This interaction results in distinct near‐surface dipole configurations depending on the bulk polarization direction (P↑ or P↓). In the P↑ state (Figure 4d), the bulk dipole (upward) gradually decreases as it approaches the subsurface layer, where it reverses to a downward dipole (Figure 4f,h). This inversion occurs progressively across several atomic layers, mitigating polarization charge buildup and reducing near‐surface polarization in the P↑ state. Conversely, in the P↓ state (Figure 4c), the downward subsurface dipole integrates seamlessly with the bulk dipole (also downward), forming a uniform dipole orientation extending up to the subsurface layer (Figure 4e,g). This dipole configuration eliminates the need for internal dipole inversion, allowing the P↓ state to retain a greater proportion of the bulk polarization up to the surface.

The stabilization of the downward subsurface dipole is closely linked to the planar accumulation of V_O_ in the subsurface layer.^[^ ^31^ ^]^ The atomic relaxation around V_O_ can be understood through the Coulombic interaction between the positively charged V_O_ centers and neighboring cations and oxygen atoms, in which cations are repelled, and oxygen atoms are attracted. Previous studies have shown that this interaction induces an upward dipole above the V_O_ and a downward dipole below it, forming a tail‐to‐tail dipole configuration.^[^ ^31^, ^32^ ^]^ Planar accumulation of V_O_ in the subsurface layer can further minimize both the area and energy of a tail‐to‐tail domain boundary.^[^ ^31^, ^33^ ^]^ Additionally, the positive charges localized at V_O_ centers effectively compensate for the negative bound charges at the tail‐to‐tail domain boundary. Thus, the planar accumulation of V_O_ in the subsurface layer, aligned perpendicular to the polarization axis, is crucial for stabilizing the tail‐to‐tail dipole configuration. It is important to note that for this V_O_‐stabilized tail‐to‐tail dipole configuration, the P↓ state is energetically favored over the P↑ state. In the P↓ state, the compensation of the depolarization field occurs without the additional energy required for the gradual reduction and rotation of polarization, making it a more stable configuration.

### 2.5 Theoretical Calculation

To investigate the effects of subsurface V_O_ on the depolarization of BaTiO_3_ (001) surface in P↑ and P↓ states, we conducted the first‐principles density functional theory (DFT) calculations. The polarization of bulk BaTiO_3_ is known to be ≈30 µC cm^−2^. If the polarization persists to the (001) surface without compensation, it would deposit surface charges with a density of ≈0.4*e* per u.c. The DFT calculations show that the presence of a single V_O_ in the subsurface layer of a $\sqrt{5}\times \sqrt{5}$ BaTiO_3_ supercell slab can fully compensate for these surface charges (Figure S10, Supporting Information). We note that our study focuses on neutral V_O_, as they represent the most relevant defect type for the polar BaTiO_3_ surface; neutral V_O_ is generally stable across most of the allowed Fermi level range for polar BaTiO_3_, whereas charged V_O_ is stabilized only under specific conditions near the valence band maximum.^[^ ^62^ ^]^ As to the (001) surface structure of the BaTiO_3_ supercell, we used a simplified surface structure with a (1 × 1) BaO termination, ignoring TiO_x_ adunits. Despite this simplification, the model accurately reproduces the key features of the surface structure, particularly the tail‐to‐tail rumpling pattern, demonstrating that subsurface Vo is a dominant factor governing atomic rumpling, as discussed in detail below. It is important to note that the same BaTiO_3_ supercells without V_O_ or with V_O_ but in the surface layer could not reproduce the tail‐to‐tail rumpling pattern observed in the experiment after relaxation (Figures S11,S12, Supporting Information).

**Figure 5:**
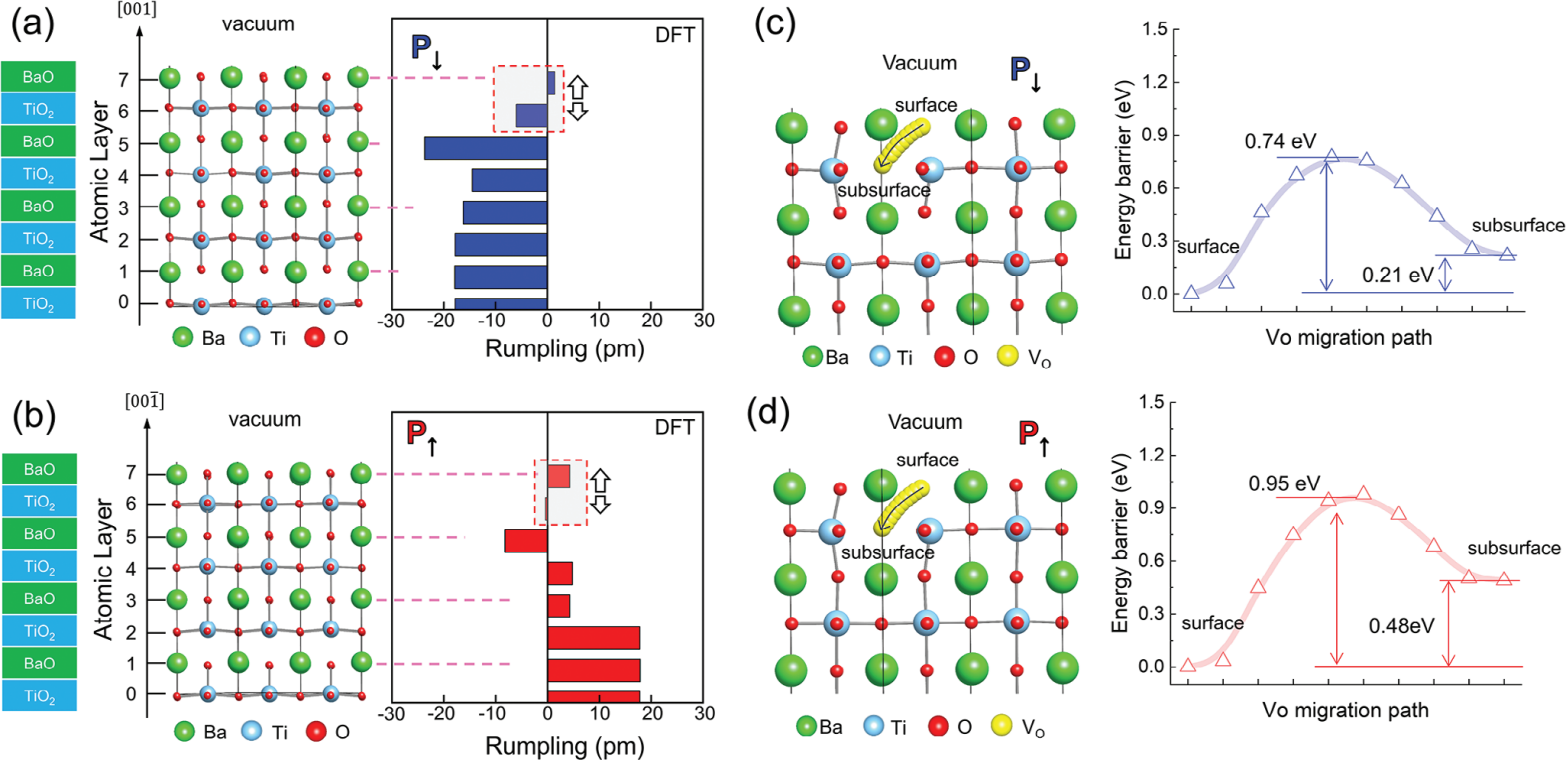
DFT calculation on atomic rumpling and oxygen vacancy (V_O_) migration in a model BaTiO_3_ (001) surface. Atomic rumpling of BaO‐terminated (1 × 1) BaTiO_3_ (001) surface in P↓ (a) and P↑ (b) state. The atomic layers are numbered sequentially from the bulk to the surface, with even‐numbered layers corresponding to TiO_2_ and odd‐numbered layers corresponding to BaO. In this sequence, layer 7 represents the topmost BaO surface layer, while layer 6 corresponds to the underlying TiO_2_ subsurface layer. V_O_ is introduced to the subsurface layer (layer 6). The rumpling amplitude is sequentially plotted for each BaO and TiO_2_ layer. The tail‐to‐tail dipole inversion in the surface and subsurface layers is observed in both P↓ and P↑ states only when V_O_ exists in the subsurface TiO_2_ layer. For comparison, refer to the rumpling patterns from the same surface structure without V_O_ in the surface layer (Figure S11, Supporting Information) and with V_O_ in the surface layer (Figure S12, Supporting Information). DFT simulations for the migration barrier of V_O_ from the surface to the subsurface layer in the P↓ (c) and P↑ (d) states. The possible V_O_ migration path is depicted by yellow circles. The energy landscape along the migration pathway of V_O_ is plotted with respect to the migration coordinates for P↓ (c) and P↑ (d) states. The smaller energy difference between the surface and the subsurface configurations in the P↓ state indicates a relatively higher density of V_O_ in the subsurface layer compared to the P↓ state.

**Figure** **5**a shows the fully relaxed atomic structure of the BaTiO_3_ slab in the P↓ configuration after introducing a V_O_ into the subsurface TiO_2_ layer, along with the resulting rumpling pattern. The atomic layers are numbered sequentially from the bulk to the surface, with even‐numbered layers representing TiO_2_ and odd‐numbered layers representing BaO. In this sequence, layer 7 corresponds to the topmost BaO surface layer, and layer 6 represents the underlying TiO_2_ subsurface layer. A tail‐to‐tail rumpling configuration is induced by the presence of Vo in the subsurface TiO_2_ layer, indicating significant Coulombic interactions between the positively charged V_O_ and surrounding atoms. As reported previously, this interaction induces upward and downward rumpling above and below the V_O_, respectively, stabilizing the tail‐to‐tail dipole configuration. Importantly, this configuration consistently forms in both the P↓ and P↑ states (Figure 5a,b), strongly suggesting that the presence of V_O_ is the key driving factor for this formation. In the P↓ state, the downward subsurface rumpling aligns fully with the bulk rumpling, resulting in all rumplings sharing the same sign (orientation) up to the subsurface, with only a gradual change in magnitude. Conversely, in the P↑ state, the downward subsurface rumpling opposes the bulk rumpling, necessitating the occurrence of sign reversal below the subsurface layer. For that reason, the P↑ state with Vo in the subsurface TiO_2_ layer is energetically less favorable than the P↓ state.

To confirm the stability of Vo in the subsurface TiO_2_ layer, we examined the migration of Vo from the surface to the subsurface layer. We considered the surface‐to‐subsurface migration of Vo to account for the observed density of Vo in the subsurface TiO_2_ layer rather than direct Vo formation in the subsurface, as the former costs less energy. Figure 5c,d depict the migration pathway for Vo from the BaO surface to the subsurface TiO_2_ layer, along with the associated energy barriers in the P↓ and P↑ states, respectively. The formation energy of Vo in the subsurface layer for the P↑ state is ≈0.48 eV higher than in the surface layer. In contrast, in the P↓ state, the formation energy is only ≈0.21 eV higher in the subsurface than in the surface layer. Additionally, Vo migration to the subsurface requires 0.95 eV in the P↑ state, compared to only 0.74 eV in the P↓ state. These results confirm that the P↑ state with Vo in the subsurface TiO_2_ layer is energetically less stable than the P↓ state, which explains why the P↓ state with a dense distribution of Vo is frequently observed in our experiments. Moreover, when V_O_ is introduced into the subsurface TiO_2_ layer, our layer‐by‐layer projected density of states calculations reveal no significant potential drop across the BaO and TiO_2_ layers in the BaTiO_3_ slab, regardless of whether the polarization is in P↓ or P↑ state (Figure S10, Supporting Information). This strongly suggests that the subsurface Vo can fully stabilize the ferroelectric BaTiO_3_ (001) surface. Given that GGA functionals can underestimate charge localization and binding energies in oxide systems, we performed supplementary calculations using a hybrid functional to improve the accuracy of the Vo migration barrier heights. These calculations confirmed that the overall trends and conclusions remain consistent with our original results (Figure S13, Supporting Information). We also investigated the effect of van der Waals (vdW) corrections on the atomic rumpling in the P↓ or P↑ states, as well as on the Vo migration barrier height. Incorporating vdW corrections resulted in minor adjustments to the rumpling amplitude (Figure S14, Supporting Information) and Vo migration barrier height (Figure S15, Supporting Information).

Our theoretical calculations not only confirm the energetic stability of Vo in the subsurface layer but also demonstrate that these Vo pin the surface and subsurface rumpling, independent of the bulk polarization direction. The resulting tail‐to‐tail dipole configuration uniquely stabilizes the asymmetry in the double‐well potential, effectively breaking the degeneracy between P↓ and P↑ states in the presence of subsurface Vo in the BaTiO_3_ (001).

## 3 Conclusion

The in‐situ atomic‐scale imaging and spectroscopy conducted under typical annealing conditions for oxide surfaces revealed that the BaTiO_3_ (001) surface stabilizes into a (2 × 1) BaO‐terminated reconstruction, accommodating BaO deficiency and incorporating TiO_x_ adunits. These TiO_x_ adunits remain stable down to room temperature although the (2 × 1) surface symmetry becomes less pronounced. The surface layer exhibits atomic rumpling, where cations are displaced above oxygen atoms in both P↑ and P↓ states, generating an upward surface dipole. Beneath this, the subsurface TiO_2_ layer plays a crucial role in accommodating V_O_, thereby compensating the depolarization field. The planar accumulation V_O_ in the subsurface TiO_2_ layer induces a downward dipole, which is consistent in both the P↑ and P↓ states.

The cooperative atomic rumpling of the surface and subsurface layers, arranged in a tail‐to‐tail configuration and stabilized by V_O_ accumulation in the subsurface TiO_2_, effectively reduces the net polarization of surface unit cells, contributing to overall depolarization. Under this unique atomic rumpling, the P↓ state is favored over the P↑ state, as the former requires less extensive atomic relaxation in the bulk layers to achieve dipole inversion at the subsurface layer. The energetic preference for V_O_ in the subsurface TiO_2_ of the P↓ state was confirmed through DFT calculations of V_O_ formation energy and the energy barrier for the surface‐to‐subsurface migration. These findings reveal that the presence of Vo in the subsurface layer lifts the degeneracy in the double‐well potential between P↓ and P↑ state in BaTiO_3_ (001).

This work highlights the crucial role of the subsurface layer in surface reconstruction and depolarization processes of ferroelectric or polar oxide surfaces. Specifically, it demonstrates how the accommodation of V_O_ and the induced atomic rumpling (or buckling) in the subsurface layer help counteract bulk polarization. These findings suggest a broader principle: at elevated temperatures, where entropy becomes a dominant factor, surface reconstruction may extend beyond the outermost layer into the subsurface through dynamic V_O_ exchange and associated atomic rearrangements.

Insights from these observations suggest a new strategy for miniaturizing ferroelectric devices with overcoming the surface depolarization effects. The stabilization mechanisms revealed in this study, particularly the role of subsurface V_O_, offer a new pathway for designing single‐unit‐cell‐thickness ferroelectrics where tail‐to‐tail dipole configuration is stabilized through planar accumulation of V_O_ in the middle of the unit cell. Such structures can be used to develop high‐density memory cells with improved reliability and tunable polarization states. These advancements enable nanoscale ferroelectric components to operate efficiently under extreme conditions, underscoring the importance of in‐situ, depth‐resolved, atomic‐scale characterization for advancing surface science.

## 4 Experimental Section

### TEM Sample Preparation

TEM specimens with the (001) surface in edge‐on projection were prepared along the [100] and [110] zone‐axes of a BaTiO_3_ single crystal (CrysTec GmbH). FIB milling was performed using a Helios G4 instrument (FEI), with a final low‐energy Ga^+^ ion beam polishing at 2 kV to reduce the beam‐induced damages. The FIB lamella was mounted on a Si MEMS chip (Wildfire, DENSsolutions) for in situ heating experiments. To ensure a clean BaTiO_3_ (001) surface, the lamella was annealed in the high vacuum of the TEM column (≈2 × 10^−5^ Pa) at 1000–1100 °C for 10 min.

### In‐Situ HRTEM Imaging

In‐situ heating experiments were performed using a double Cs‐corrected field‐emission TEM (JEM‐ARM300F, JEOL) operating at 300 kV. HRTEM images and movies were recorded under a negative spherical aberration imaging (NCSI) condition where all atomic columns of BaTiO_3_, including oxygen, were resolved clearly. Before image acquisition, the objective lens aberrations were evaluated using the Zemlin tableau method from a thin amorphous region near the edge of TEM specimens.^[^ ^63^ ^]^ After iterative tuning, the spherical aberration was finely tuned to −16–−13 µm, ideal for NCSI, providing high‐contrast images with minimal contrast delocalization (≈±40 pm), enabling precise determination of atomic column positions within a picometer precision (±4 pm).^[^ ^60^, ^64^ ^]^ Defocus ranged from +4 to +6 nm, with A1 below 1 nm, A2 under 25 nm, and B2 below 10 nm. The electron dose rate was maintained at ≈1 × 10^4^ e⁻ Å⁻^2^ s⁻¹. Real‐time movies were captured at 25 frames per second using a 4096 × 4096‐pixel CMOS camera (OneView, Gatan).

### HRTEM Image Analyses

Rigid registration was applied iteratively during the post‐processing of in‐situ HRTEM movies to minimize image drift. An inner bulk region with stable contrast was selected as the reference for registration, reducing the measured drift to be less than 10^−5^ nm. This process ensured accurate tracking of atomic column positions throughout the experiment, particularly during surface evaporation.

Post‐processing of the image sequences was performed using TrackMate in ImageJ and custom scripts developed for DigitalMicrograph (Gatan). The Center‐of‐Mass (CoM) method was employed to precisely calculate the center positions of Ba, TiO, and O atomic columns along the [100] zone axis, as well as BaO, Ti, and O columns along the [110] zone axis. Peak picking by CoM allowed for high‐precision determination of column positions based on the intensity values from each frame.

During surface evaporation, 3‐frame averaging was applied to improve the signal‐to‐noise ratio (SNR) while maintaining temporal resolution, enabling accurate tracking of intensity modulations and analysis of atom movements to freshly exposed surfaces during evaporation. For polarization mapping at the BaTiO_3_ surface, 10‐frame averaging was applied to further enhance the SNR, resulting in highly precise intensity measurements. No image filters were applied to avoid introducing artifacts, ensuring the accuracy of both position and intensity measurements.

### HRTEM Image Simulation

100HRTEM image simulations were carried out using the abTEM Python package to determine the imaging condition of experimental NCSI HRTEM images and, more importantly, the thickness of the TEM specimen.^[^ ^65^ ^]^ As the intensity of the oxygen column was linearly proportional to the number of atoms in the corresponding column under NCSI conditions, accurate determination of TEM sample thickness was crucial for analyzing oxygen vacancies. As illustrated in Figures S7 and S8 (Supporting Information), it was confirmed that the linear intensity‐thickness relationship was valid when the sample thickness remained smaller than 6 u.c. To match the simulated images with experimental data, the modulation transfer function of the CMOS camera (OneView, Gatan) operating at 300 kV was considered, addressing the Stobbs‐factor issue, which could otherwise distort the comparison between simulated and experimental results. Initial parameters (spherical aberration (Cs) and defocus) were determined via cross‐correlation with experimental data using an iterative digital image‐matching process.^[^ ^66^ ^]^ A thickness‐defocus map was generated for the tetragonal BaTiO_3_ structure along the [100] zone axis to refine the sample thickness. The measurement precision was further verified by comparing simulated and experimental images by performing image cross‐correlation across multiple defocus conditions. Once the thickness was determined for the TiO columns in the TiO_2_ atomic layer, the expected intensity of the O column in the simulation was compared with those in the experimental images to determine the presence of oxygen vacancy.

### Polarization Measurement using HRTEM Images

In NCSI HRTEM images, the positions of individual atomic columns were determined by locating the CoM of the column intensities through an iterative method. The center of each unit cell was defined using the position of the A‐site cations (Ba), and the displacement of the B‐site cations (*δ*_Ti_) was subsequently derived. The center of the oxygen octahedral cage was approximated by the positions of two equatorial and two apical oxygen anions, and the oxygen displacement (*δ*_O_) from the unit cell center was determined. The in‐plane lattice parameter (*a*) and out‐of‐plane lattice parameter (*c*) for the tetragonal structure of BaTiO_3_ at room temperature were measured and used to calculate the ferroelectric polarization. The polarization (*P*) was calculated using the following formula:

(1) $$P=\frac{e}{V}\sum_{i}\delta_{i}Z_{i}=\frac{e}{V}\left(\delta_{Ti}Z_{Ti}+3\delta_{O}Z_{O}\right)$$

where *e* is the charge of the electron, *V* is the volume of the unit cell, and *Z_i_* is the ionic charge of the *i* atom. In this study, the following formal charges of: *Z_Ti_* = 4; *Z_O_* = −2 were used.

### EELS Data Acquisition

Aberration‐corrected STEM (JEM‐ARM300CF, JEOL), equipped with an energy filter (Gatan Quantum ER965), was used to analyze the elemental composition and electronic structure of BaTiO_3_. For elemental mapping, EELS 2D array data from the BaTiO_3_ surface were recorded over an energy range of 375–875 eV, covering the Ba‐M_4,5_, Ti‐L_2,3_, and O‐K edges. The energy dispersion and resolution were set to 0.25 eV pixel^−1^ and 1.25 eV, respectively. For the fine structure analysis, EELS 2D array data were recorded in the 413–612 eV range, focusing on the Ti‐L_2,3_ and O‐K edges, with an energy dispersion of 0.1 eV pixel^−1^ and a resolution of 0.6 eV. The dwell time for both elemental mapping and fine structure analysis was set to 0.02 s to minimize specimen drift and prevent electron beam damage. The electron dose rate was maintained at ≈10^6^ e^−^ nm^−2^ s^−1^, ensuring no significant damage during data collection.

### Analysis of Ti Valence State

Energy loss near edge structure was employed for the quantitative analysis of Ti ion valence states. In BaTiO_3_, Ti ions typically exhibit a 4+ valence state without additional electrons in the 3*d* orbital. When excess electrons occupy the Ti 3*d* orbital, the density of states available for core electron excitation decreases, altering the fine structure of the core‐loss spectra. To quantify these changes, reference spectra for Ti^3+^ and Ti^4+^ were obtained from LaTiO_3_ and BaTiO_3_, respectively.^[^ ^59^ ^]^ Experimental EEL spectra were deconvoluted using MLLS fitting, which assumed the measured spectrum was a linear combination of the reference spectra with variable fractions. While MLLS fitting yields quantitative estimates of the valence state at each probe position, it did not account for dynamic scattering or channeling effects, which might introduce spatial blurring in the valence state profiles.

### EDS Data Acquisition

STEM images were acquired using a cold field‐emission (S)TEM (JEM‐ARM200CF, JEOL) with double Cs‐correctors, operated at 200 kV. STEM‐EDS data were also collected at 200 kV using double silicon drift detectors with a total detector area of 200 mm^2^ and a solid angle of 2.4 sr (JEOL). A custom Python script was employed to correct specimen drift and align the EDS mapping data, with additional data processing performed using non‐local PCA. For the elemental maps, characteristic X‐ray peaks of Ba‐Lα and Ti‐Kα were deconvoluted based on the ratio of Ba‐Lα to Lβ to obtain the quantitative composition of Ba and Ti.

### DFT Simulation

DFT calculations were performed using the Vienna ab initio simulation package^[^ ^67^ ^]^ and the projector‐augmented wave method of Blochl^[^ ^68^ ^]^ in the implementation of Kresse and Joubert. The Perdew–Burke–Ernzerhof generalized gradient approximation (GGA) was employed as the exchange‐correlation functional.^[^ ^69^ ^]^ A plane wave basis set with an energy cutoff of 600 eV was employed, and a Γ‐centered 4 × 4 × 1 k‐point mesh was used for the supercell slabs. The convergence criteria for the electronic relaxation were set to 10^−6^ eV cell^−1^, and the structures were relaxed until the forces were below 0.05 eV Å^−1^. To calculate the rumpling in BaTiO_3_, a $\left(\sqrt{5}\times \sqrt{5}\right)$ BaTiO_3_ slab with 20 Å of vacuum was constructed to minimize boundary effects. Symmetrical termination of the BaTiO_3_ surfaces (P_↑_ and P_↓_) with a BaO layer was employed, while the central five layers (two BaO and three TiO_2_) were fixed in their bulk configurations. Dipole corrections were applied to eliminate the artificial electric field in the vacuum region. Additionally, the contributions of vdW interactions and hybrid functional corrections to surface reconstruction and oxygen vacancy migration were analyzed. In this work, the vdW interaction was treated by the Grimme D3 method^[^ ^69^ ^]^ and the hybrid functional was applied using the HSE06.^[^ ^70^, ^71^ ^]^ These results revealed negligible changes in rumpling amplitudes and migration barriers (Figures S13–S15, Supporting Information), confirming that vdW interactions and hybrid functional corrections had no significant effects on the qualitative trends of the studied phenomena. Therefore, these contributions were not further considered in the main analysis. The DFT calculation was performed by using high‐performance computing clusters in the quantum matter core‐facility of Pusan National University.

## Conflict of Interest

The authors declare no conflict of interest.

## Author Contributions

J.J. and J.H. contributed equally to this work.

## Supporting information



Supporting Information

Supplemental Movie 1

Supplemental Movie 2

Supplemental Movie 3

Supplemental Movie 4

## Data Availability

The data that support the findings of this study are available from the corresponding author upon reasonable request.
