# Supplementary material for: Subsurface Oxygen Vacancy Mediated Surface Reconstruction and Depolarization of Ferroelectric BaTiO3 (001) Surface
Source: Adv Sci (Weinh). 2025 Feb 13;12(16):2412781. doi: 10.1002/advs.202412781 (PMC12021061; doi:10.1002/advs.202412781)
Supplement: Supplementary file 1 — Supporting Information [file ADVS-12-2412781-s003.pdf]

ADVANCED SCIENCE
Open Access

Supporting Information

for *Adv. Sci.*, DOI 10.1002/advs.202412781

Subsurface Oxygen Vacancy Mediated Surface Reconstruction and Depolarization of Ferroelectric $BaTiO_3$ (001) Surface

Jeehun Jeong, Jaejin Hwang, Yaolong Xing, Zhipeng Wang, Jaekwang Lee and Sang Ho Oh**

Supporting Information

Subsurface Oxygen Vacancy Mediated Surface Reconstruction and Depolarization of Ferroelectric $BaTiO_3$ (001) Surface

Jeehun Jeong, Jaejin Hwang*, Yaolong Xing, Zhipeng Wang, Jaekwang Lee, Sang Ho Oh*

J. Jeong, Y. Xing, Z. Wang, S. H. Oh

Department of Energy Engineering, KENTECH Institute for Energy Materials and Devices, Korea Institute of Energy Technology (KENTECH), Naju 58330, Republic of Korea.

J. Hwang, J. Lee

Department of Physics, Pusan National University, Busan 46241, Republic of Korea.

E-mail: jaekwangl@pusan.ac.kr

S. H. Oh

Center for Shared Research Facilities, Korea Institute of Energy Technology (KENTECH), Naju 58330, Republic of Korea.

E-mail: shoh@kentech.ac.kr

Movie Legends

Movie S1. In-situ HRTEM movie showing $BaTiO_3$ surface evaporation at 1000 °C. HRTEM movie, captured under a negative Cs imaging (NCSI) condition at 1000 °C, shows the layer-by-layer evaporation of the $BaTiO_3$ (001) surface, with an atomic step advancing over time. To enhance the contrast, every 3 consecutive frames from the original 25 frames per second (fps) recording were averaged. After the atomic step moved away, the freshly exposed BaO-terminated surface exhibits a (2×1) reconstruction as delineated by white lines.

Movie S2. In-situ HRTEM movie and atomic column intensity analysis at [100] zone axis showing the atomic step assisted non-stoichiometric (2×1) reconstruction process. A time-series intensity measurement of the TiO (blue) and O (orange) columns during the evaporation $BaTiO_3$ surface at 1000 °C. The decrease of TiO column intensity upon removal by step migration is correlated with the intensity rise of O column in the BaO layer which is exposed as a new surface. This coupled intensity change indicates the diffusion of TiO_x from the moving step edge toward the surface, where it arranges into stable (2×1) reconstruction. The zone axis is [100]. To enhance clarity, every 5 frames were averaged, and intensity peaks were measured within a 7-pixel (~40 pm) radius.

Movie S3. 2. In-situ HRTEM movie and atomic column intensity analysis at [110] zone axis showing the atomic step assisted non-stoichiometric (2×1) reconstruction process. A time-series intensity measurement of the Ti (blue), O (green), and octahedral interstitial (O_h) site (orange) during the evaporation $BaTiO_3$ surface at 1000 °C. The decrease of Ti column intensity upon removal by step migration is correlated with the intensity rise of vacant octahedral (O_h) sites between Ba columns O column in the BaO layer which is exposed as a new surface. This coupled intensity change indicates the diffusion of TiO_x from the moving step edge toward the surface, where it arranges into stable (2×1) reconstruction. The zone axis is [110]. To enhance clarity, every 5 frames were averaged, and intensity peaks were measured within a 7-pixel (~40 pm) radius.

Movie S4. In-situ HRTEM movies showing the evolution of a (2×1) surface reconstruction of clean, vacuum-annealed $BaTiO_3$ with temperatures. A series of HRTEM movies captures from a clean, atomically smooth BaO-terminated surface of $BaTiO_3$ after annealing at 1000 °C. The surface is imaged along the [100] zone axis at 1000 °C, 700 °C, 500 °C, and 300 °C. To enhance the visual contrast, every 3 consecutive frames from the original 8 fps were averaged. As the temperature decreased from the annealing condition, the characteristic rumpling pattern of the (2×1) reconstruction became less pronounced. At

room temperature, although Ti atoms remained at the octahedral sites in the surface layer, the (2×1) reconstruction was no longer maintained over long ranges.

Supporting Information Figures

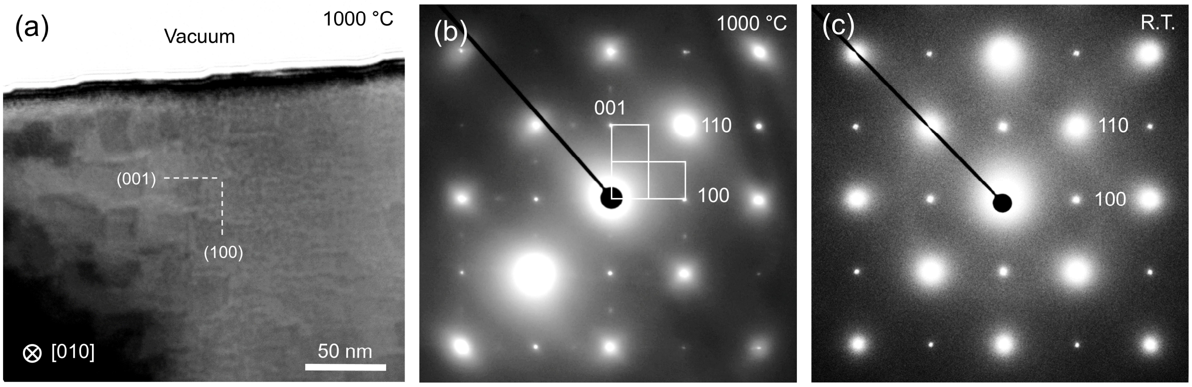

Figure S1

Surface reconstruction of $BaTiO_3$ (001) surface assessed by selected area electron diffraction pattern. (a) Bright-field TEM image of $BaTiO_3$ (010) face obtained at 1000 °C. The TEM image was recorded at an under-focus condition to enhance the step-terrace contrast by Fresnel fringes. (b) Selected area electron diffraction (SAED) pattern obtained from the $BaTiO_3$ (010) face at 1000 °C. The sample was slightly tilted off the [010] zone axis to suppress the bulk reflections. The super-reflection appearing at half of the (100) reflection indicates the two-unit cell periodicity of the surface, indicating (2×1) surface reconstruction. The existence of 90°-rotated (2×1) surface domains is indicated by white lines. (c) SAED pattern obtained from the $BaTiO_3$ (010) face at room temperature. There are no super-reflections indicative of surface reconstruction in the SAED pattern.

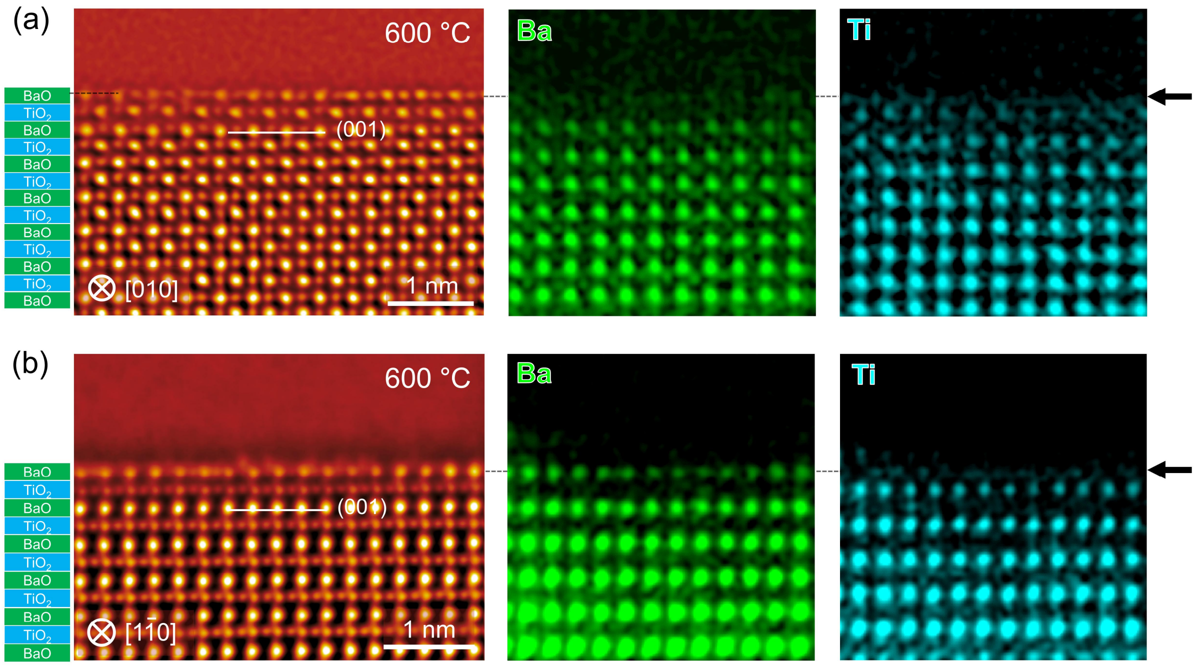

Figure S2

iDPC STEM images and EDS elemental maps showing BaO termination of $BaTiO_3$ (001) surface. Atomic-resolution iDPC STEM images and STEM-EDS elemental maps constructed by selecting Ba-Lα (deconvoluted by Ba-Lβ) and Ti-Kα characteristic X-ray acquired the [010] (a) and [110] (b) zone axis at 600 °C. Black arrows next to the Ti map indicate the surface termination layer. Existence of Ti in the BaO surface layer is noticeable.

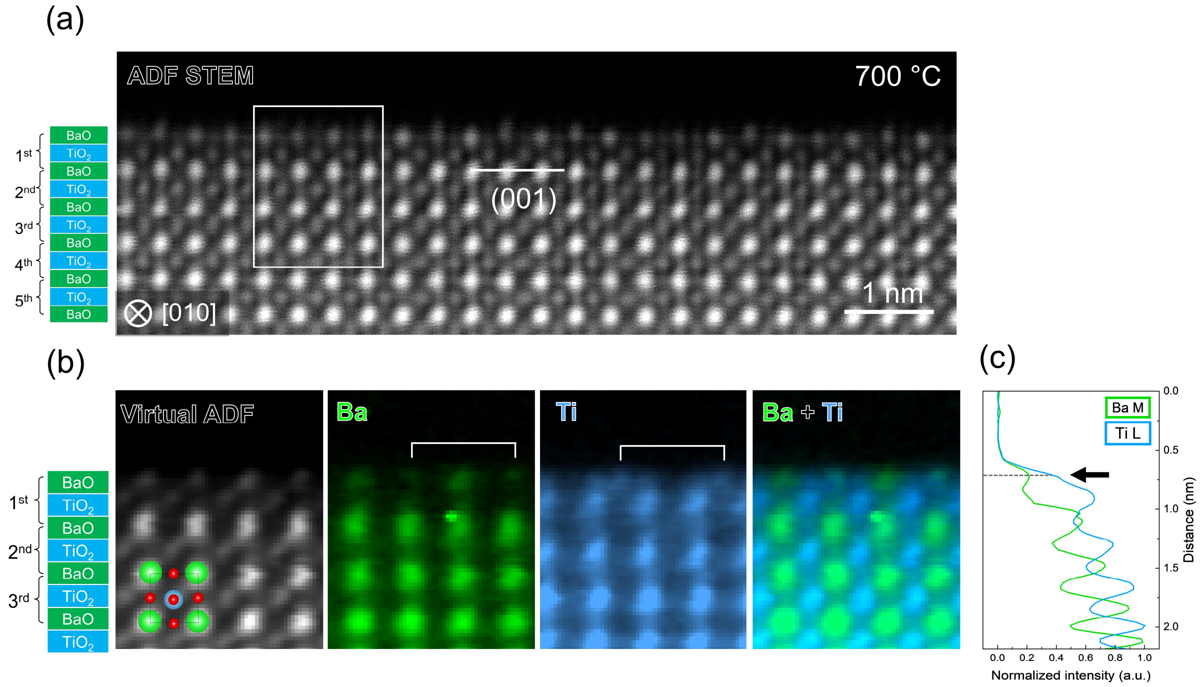

Figure S3

STEM EELS elemental maps showing the (2×1) reconstruction of BaO-terminated $BaTiO_3$ (001) surface with accommodating Ba deficiency and excess Ti. (a) ADF STEM image of $BaTiO_3$ (001) surface obtained at 700 °C. (b) A virtual ADF image and EELS elemental maps constructed by selecting EELS Ba-$M_{4,5}$ and Ti-$L_{2,3}$ edges from the region outlined in (a). The two-unit-cell periodicity of Ba deficiency and excess Ti in the BaO termination layer are indicated by white lines. (c) In-plane averaged intensity profiles of Ba and Ti signals along the surface normal. The co-existence of Ba and Ti in the surface termination layer is indicated by arrow.

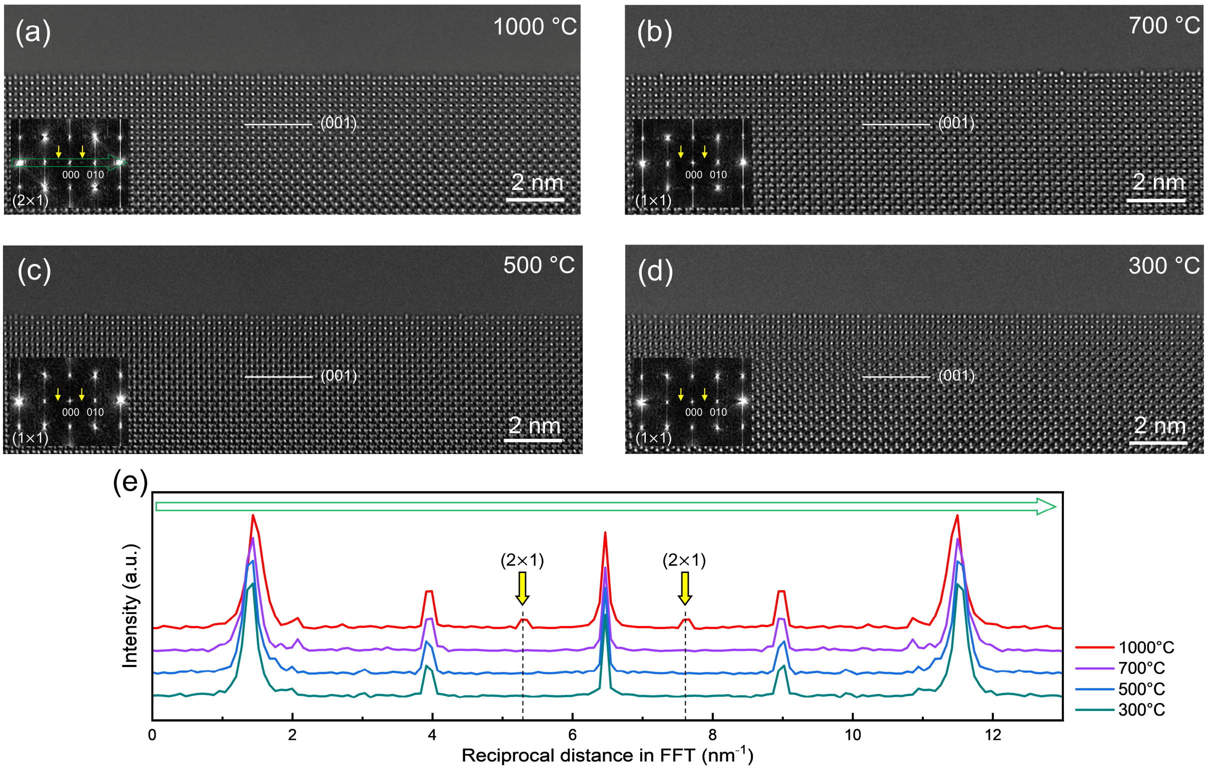

Figure S4

NCSI-HRTEM images of clean, vacuum-annealed $BaTiO_3$ (001) surfaces. HRTEM images and corresponding fast Fourier transform (FFT) patterns (insets) of $BaTiO_3$ surfaces after annealing at 1000 °C. The images were taken at 1000 °C (a), 700 °C (b), 500 °C (c) and 300 °C (d), respectively. The (2×1) surface reconstruction at 1000 °C is highlighted by red circles in the FFT pattern.

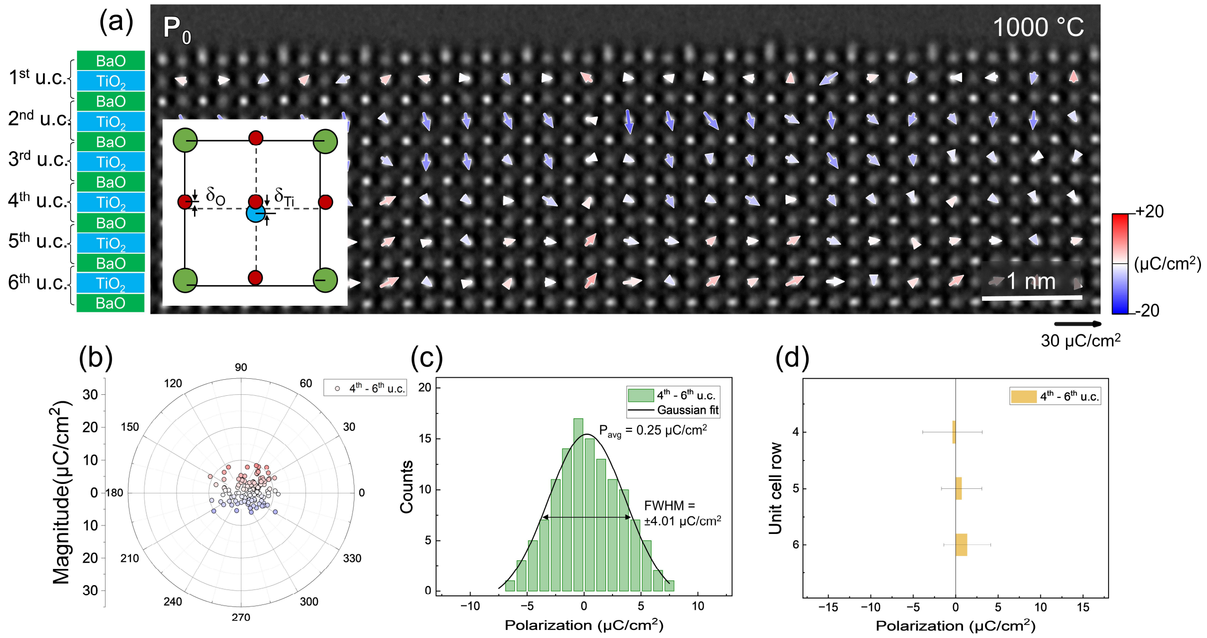

Figure S5

Method for polarization vector measurement and its precision test using a HRTEM image of cubic $BaTiO_3$. (a) NCSI HRTEM image of the (2×1) reconstructed $BaTiO_3$ (001) surface recorded at 1000 °C. As illustrated in the atomic model (inset), the displacement of Ti (δ_{Ti}) and O (δ_O) from the centrosymmetric position of Ba sublattice are measured directly on the NCSI HRTEM image. Using the measured displacements and the formal ionic charges of each element, i.e., 2+ for Ba, 4+ for Ti, and 2- for O, the polarization vector was calculated and displayed on top of TiO column of each unit cell. (b) Polar plot of the measured polarization vectors from the HRTEM image in (a). (c) Histogram of the magnitude of the polarization vectors measured from the bulk region (from the 4th to 6th u.c.). A Gaussian fit of the histogram is overlaid, from which the average value (-0.16 $\mu C\cdot cm^{-2}$) and standard deviation (±3.41 $\mu C\cdot cm^{-2}$) are determined. (d) Averaged magnitude of polarization vectors plotted for the bulk region (from the 4th to 6th u.c.). The error bars indicate standard deviation.

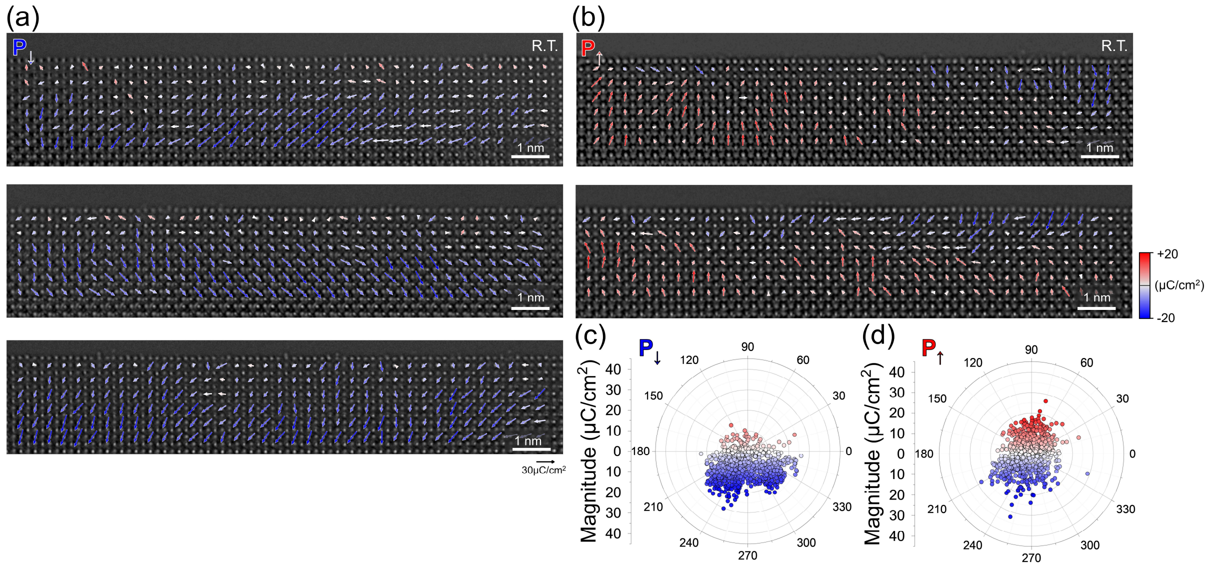

Figure S6

Selected data sets of polarization vector maps used for statistical analysis of **P**↓ and **P**↑ states. Polarization maps of tetragonal $BaTiO_3$ (001) in polarization-down **P**↓ (a) and polarization-up **P**↑ (b) state at room temperature. Polar plot of **P**↓ state (c) and **P**↑ state (d). Statistical analysis of multiple polarization maps revealed that the population of **P**↓ domains (1153 u.c.) is almost twice that of **P**↓ domains (508 u.c.).

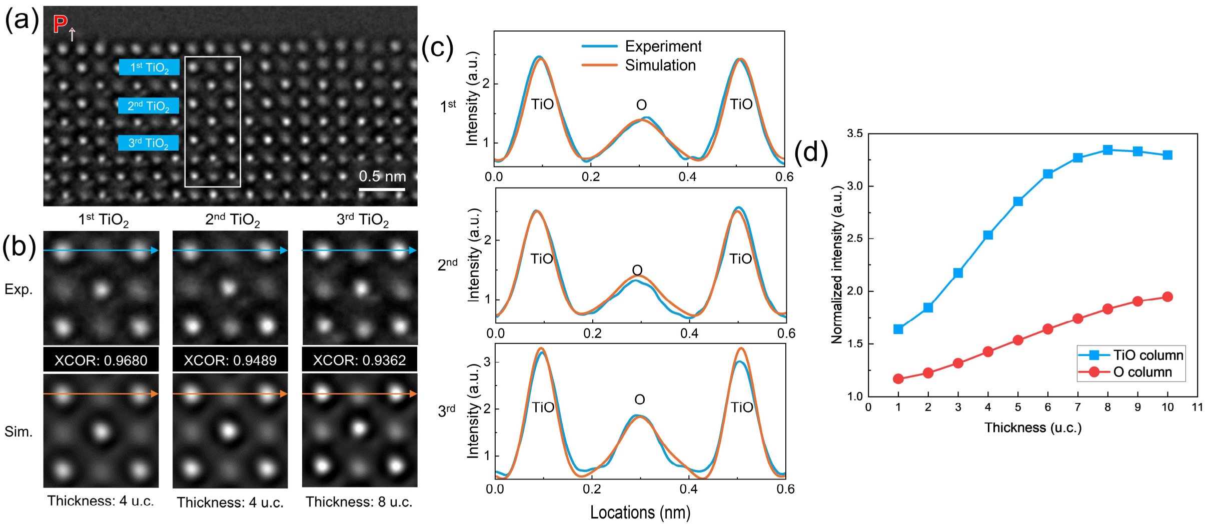

Figure S7

Measurement of oxygen vacancy in subsurface TiO_2 layers of **P**↑ state based on the intensity analysis of HRTEM image. (a) NCSI HRTEM image of $BaTiO_3$ (001) surface in **P**↑ state. (b) Magnified unit-cell images from the region outlined in (a) (upper) and simulated images (lower). From the simulated image yielding the highest cross-correlation (XCOR) factor with the experimental one the local thickness was determined. In the example, the local thickness of the 1st, 2nd and 3rd TiO_2 layer is determined to be 4 u.c., 4 u.c., and 8 u.c. respectively. (c) Intensity profiles across each TiO_2 layer obtained from experiment (blue) and simulated (orange) images. The intensity of all TiO and O atomic columns in the TiO_2 layers matches reasonably well with the simulated one, following the thickness variation. A lower intensity of the O column in the experimental image compared in that of the simulated image, which appears as uncovered light pick by dark one in Figure 2f, indicates the presence of V_O. (d) Plot of the intensity variation of the TiO and O columns in the simulated HRTEM image as a function of thickness. The linear thickness-intensity relationship up to 6 u.c. validates the use of intensity to measure the number of oxygen atoms. The image parameters used for the simulation are: Cs = -16 μm and defocus = +6 nm.

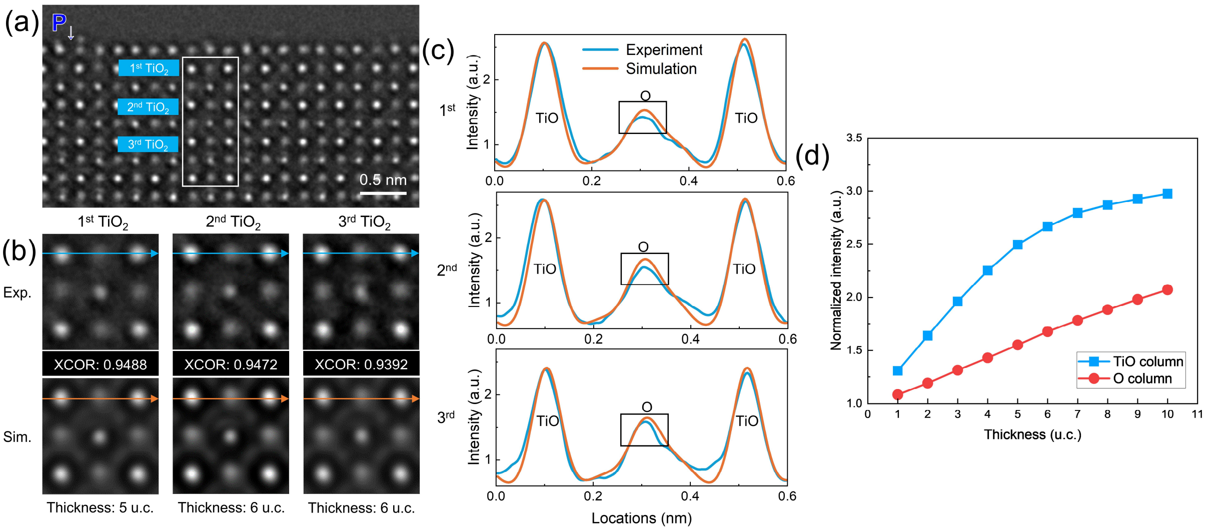

Figure S8

Measurement of oxygen vacancy in subsurface TiO_2 layers of **P↓** state based on the intensity analysis of HRTEM image. (a) NCSI HRTEM image of $BaTiO_3$ (001) surface in **P↓** state. (b) Magnified unit-cell images from the region outlined in (a) (upper) and simulated images (lower). From the simulated image yielding the highest cross-correlation (XCOR) factor with the experimental one the local thickness was determined. In the example, the local thickness of the 1st, 2nd and 3rd TiO_2 layer is determined to be 5 u.c., 6 u.c., and 6 u.c. respectively. (c) Intensity profiles across each TiO_2 layer obtained from experiment (blue) and simulated (orange) images. While the intensity of TiO columns in the TiO_2 layers matches well with the simulation, so that it is used to determine the sample thickness, the intensity of O columns is noticeably lower than the simulated one, indicating the presence of V_O in the corresponding columns. A lower intensity of the O column in the experimental image compared in that of the simulated image, which appears as uncovered light pick by dark one in Figure 2e, indicates the presence of V_O. (d) Plot of the intensity variation of the TiO and O columns in the simulated HRTEM image as a function of thickness. The linear thickness-intensity relationship up to 6 u.c. validates the use of intensity to measure the number of oxygen atoms. The image parameters used for the simulation are: Cs = -14 μm and defocus = +6 nm.

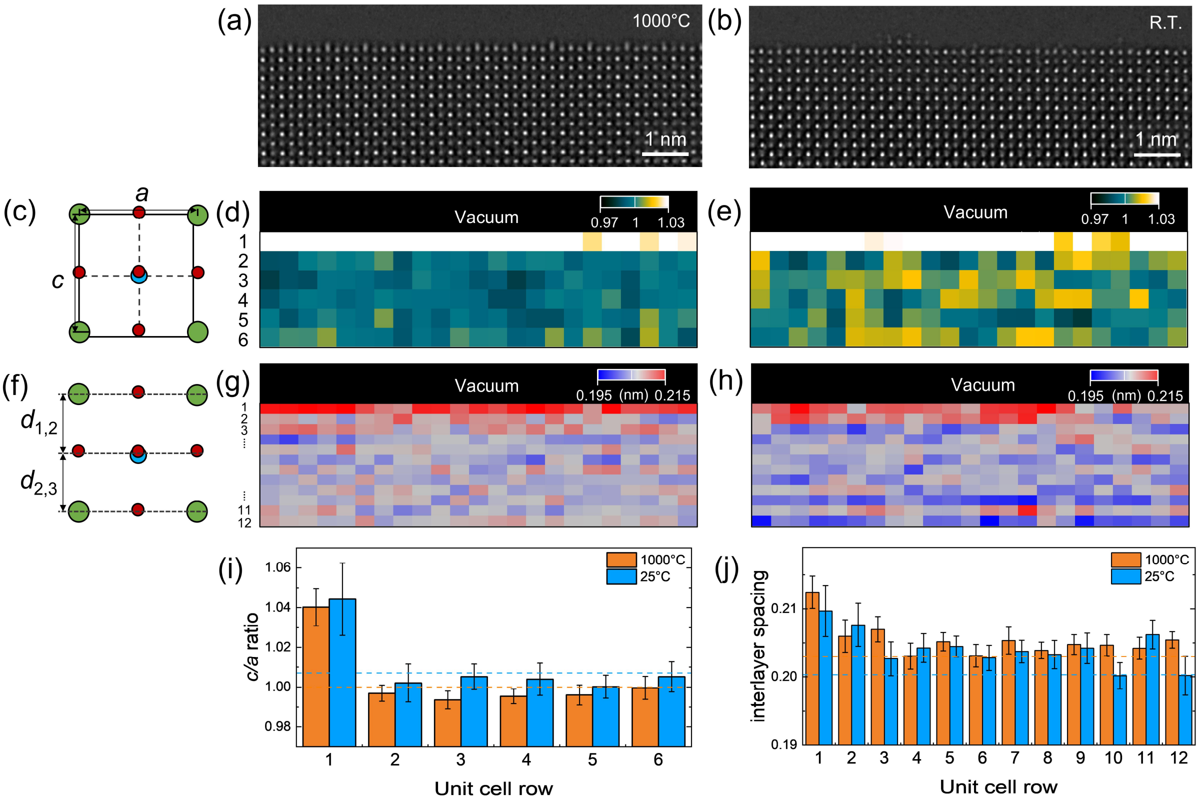

Figure S9

c/*a* ratio and interlayer spacing of $BaTiO_3$ (001) surface. NCSI HRTEM images of $BaTiO_3$ surface at 1000 °C (a) and room temperature (b). (c) Atomic model illustrating *c* and *a* lattice parameters measured from Ba columns. *c*/*a* ratio maps (colored pixels) obtained from the HRTEM recorded at 1000 °C (d) and room temperature (e). (f) Atomic model illustrating the interlayer spacing ($d_{i,j}$). Interlayer spacing maps (colored pixels) obtained from the HRTEM recorded at 1000 °C (g) and room temperature (h). (i) In–plane averaged *c*/*a* ratio of each unit cell row from the bulk to the surface. Dashed lines mark the average value of bulk region at 1000 °C (orange) and room temperature (blue). (j) In–plane averaged interlayer spacing between each layer from the bulk to the surface. The dashed lines indicate the bulk value at 1000 °C (orange) and R.T. (blue).

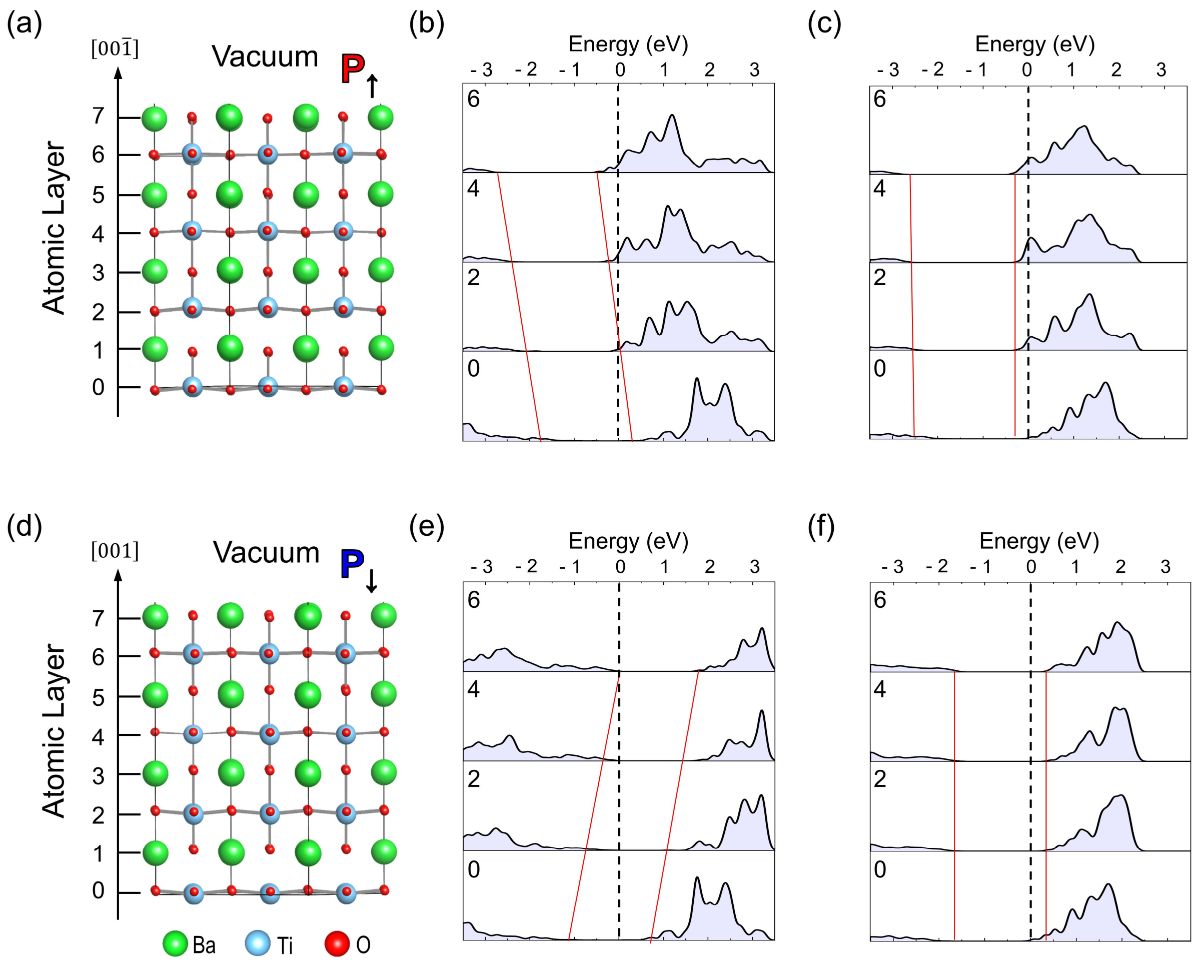

Figure S10

Relaxed atomic structure of BaO-terminated $BaTiO_3$ slab in **P**↑ (a) and **P**↓ (b) state with V_O in the subsurface TiO_2 layer. The layer-by-layer projected density of states (PDOS) in **P**↑ (c) and **P**↓ (d) state without V_O in the subsurface TiO_2 layer. PDOS in **P**↑ (e) and **P**↓ (f) state with V_O in the subsurface layer. The blue shade represents the PDOS of the Ti atoms in TiO_2 layers and the red line represents the electrostatic potential profile across the slab structures. The Fermi energy is set as zero.

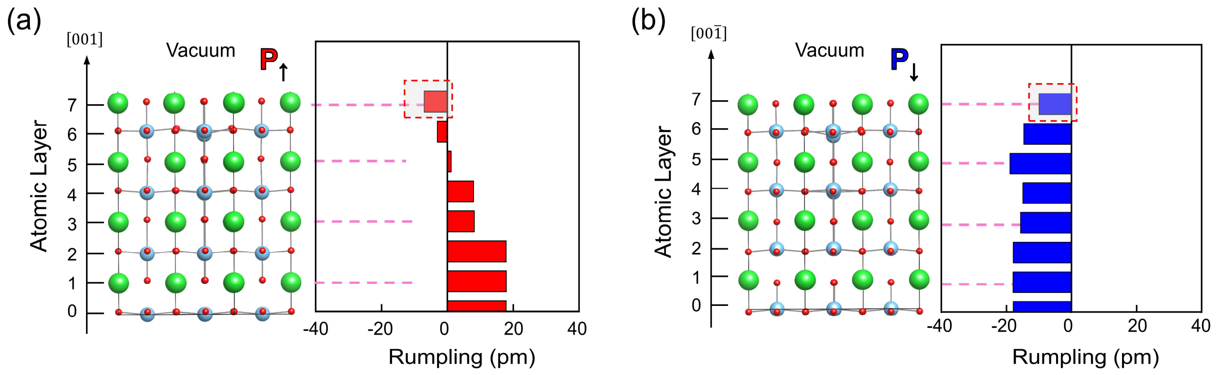

Figure S11

Relaxed atomic structures and rumpling of the $BaTiO_3$ slab in **P**↑ (a) and **P**↓ (b) state without V_O.

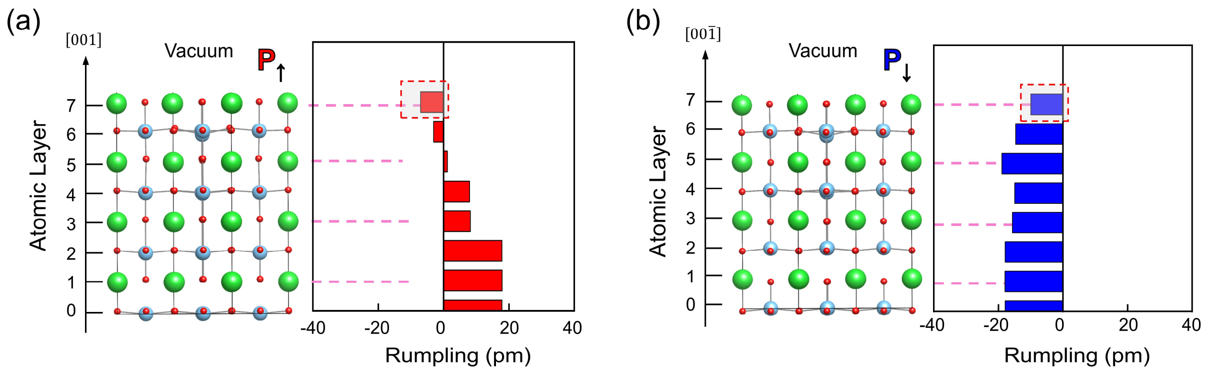

Figure S12

Relaxed structures and rumpling of the $BaTiO_3$ slab in **P**↑ (a) and **P**↓ (b) state with V_O in the surface layer.

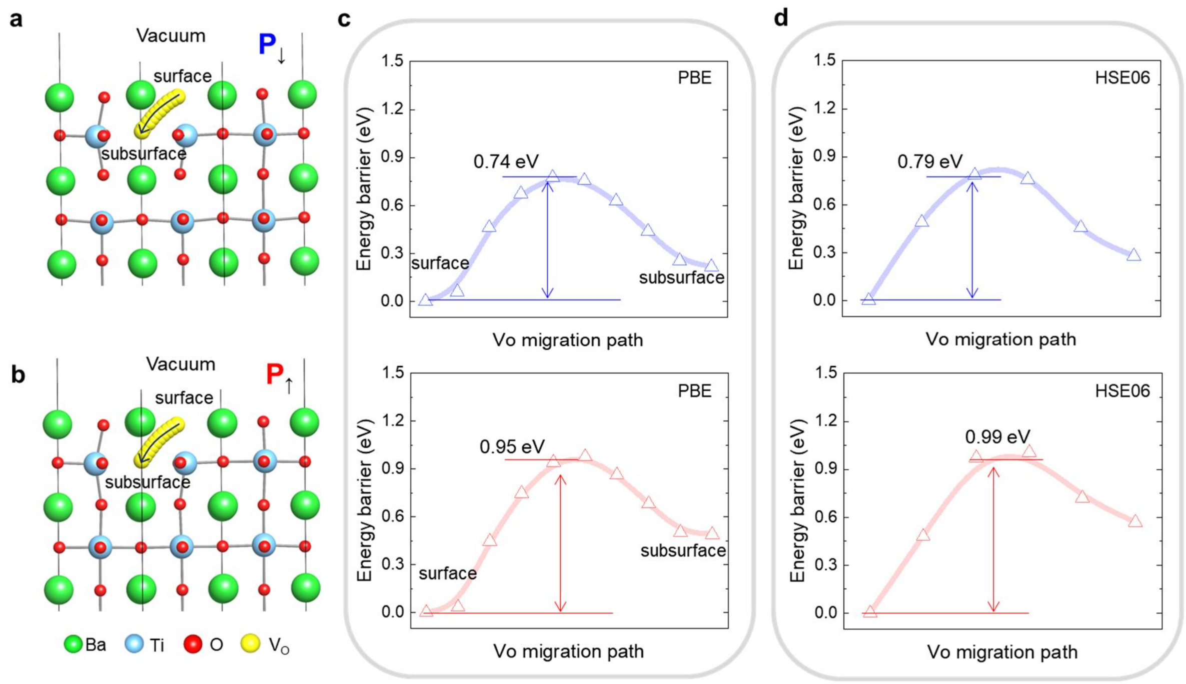

Figure S13

DFT calculation on oxygen vacancy migration in a model $BaTiO_3$ (001) surface. The Vo migration path from the BaO surface layer to the TiO_2 subsurface layer in **P**↓ (a) and **P**↑ (b) states. The barrier energies for Vo migration were calculated using the PBE exchange correlation functional (c) and hybrid exchange correlation functional (d). The energy profiles and relative trends between the **P**↓ and **P**↑ states remained unchanged, demonstrating that the choice of functional does not greatly alter the V_O migration pathway and the corresponding barrier height. Based on these observations, we conclude that PBE functional are sufficient to capture the migration pathways, the corresponding barreirs, and their dependence on polarization states.

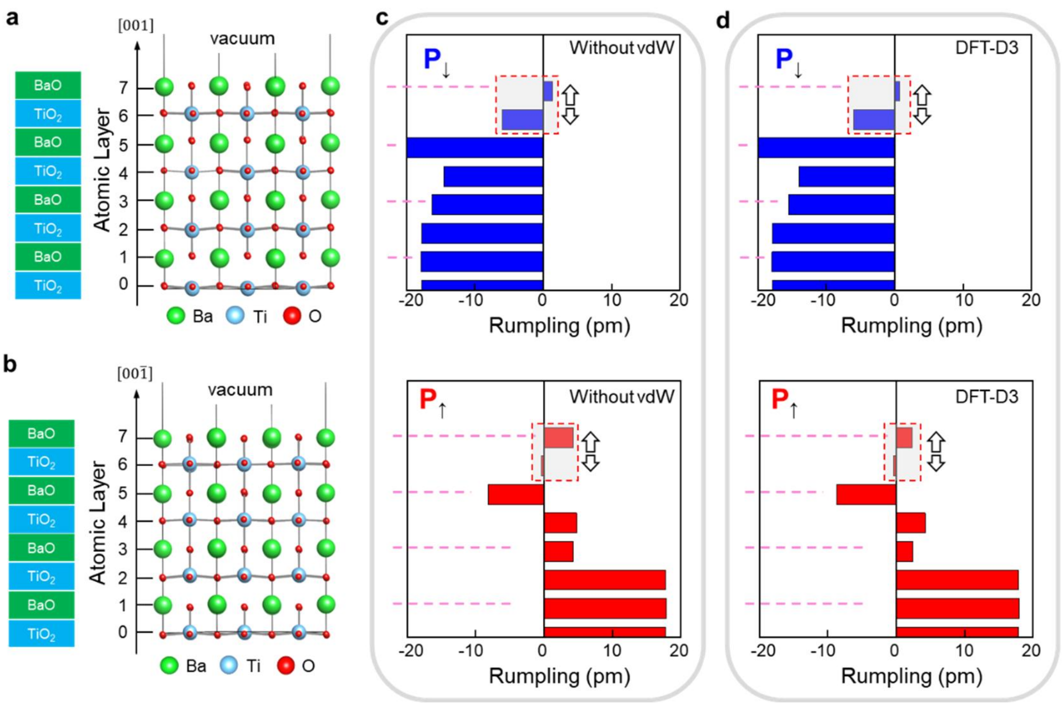

Figure S14

Effects of vdW correction on atomic rumpling in a model $BaTiO_3$ (001) surface. Side view of BaO-terminated (1×1) $BaTiO_3$ (001) surface in **P**↓ (a) and **P**↑ (b) states with Vo in the subsurface. The calculated rumpling amplitude for **P**↓ and **P**↑ states without vdW correction (c) and with vdW correction (d). The atomic rumpling amplitudes obtained using standard DFT and DFT-D3 methods are very similar. Specifically, the characteristic tail-to-tail dipole inversion in the surface and subsurface layers was consistently observed in both cases. This confirms that vdW corrections do not significantly affect the surface reconstruction of $BaTiO_3$.

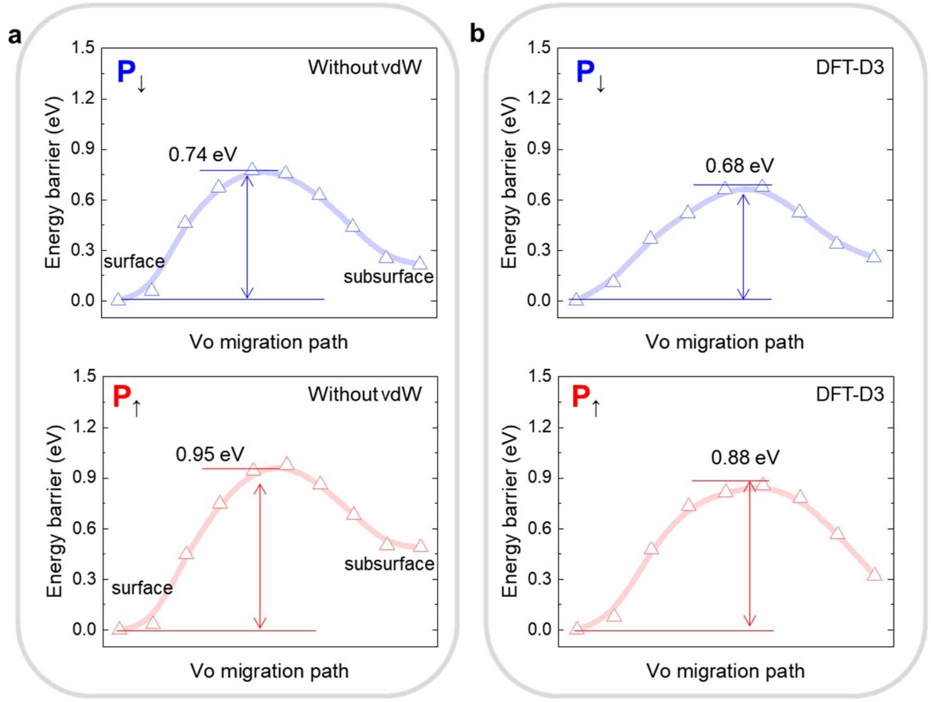

Figure S15.
Effects of vdW correction on migration of oxygen vacancy in a model $BaTiO_3$ (001) surface. The Vo migration path from the BaO surface layer to the TiO_2 subsurface layer in **P↓** (a) and **P↑** (b) states. The barrier energies for Vo migration were calculated using the standard DFT (left) and vdW correction (right). While there is a slight decrease in the barrier heights when vdW interactions are included, the overall trends remain unchanged.
